# Design and optimization of complex mechanism flip shaping subsystem based on genetic algorithm and rigid-flexible coupled dynamic model

**DOI:** 10.1371/journal.pone.0280983

**Published:** 2023-02-02

**Authors:** SHANG Xin, Yongxing LI, Xiaoxuan CHEN

**Affiliations:** School of Mechanical Engineering Dongguan University of Technology, Dongguan, Guangdong, China; University of California Santa Barbara, UNITED STATES

## Abstract

In this paper, the cam connecting rod system of the high-speed group vertical machine flipping shaping mechanism is the research object. In order to solve the key problem that the flipping shaping mechanism cannot accurately complete the action when the vibration of the mechanism is large. In this paper, the finite element method is used to construct the dynamic model of the connecting rod subsystem of the flipped shaping mechanism. And The dynamic model of cam roller subsystem is established by centralized parameter method. Based on the MATLAB Genetic Algorithm toolbox and using Newmark’s method, the dynamic equations of the flipped plastic mechanism system are solved. The optimal parameters of the connecting rod of the mechanism, the cam profile curve and the swing power and swing torque of the mechanism at different speeds are analyzed. The results show that the speed and convex contour line are important factors affecting the performance of the mechanism. And the pendulum force (swing torque) is the main cause of the vibration of the mechanism on the frame. Therefore, the mechanism pendulum dynamic and the swing moment are selected as the objective functions of the optimization model. By selecting the node parameters of the sixth order spline motion law and the cross-section parameters of the connecting rod as the design variables. The cam linkage system is optimally designed to obtain the optimal value. Finally, the optimal design of the flipped shaping mechanism was analyzed and compared with the original mechanism.

## 1 Introduction

The assembly machine is an important equipment for the production of capacitors. The surge in demand for capacitors makes it urgent to increase the speed of the device. However, high-speed operation will cause vibration and noise to the components, which will affect the accuracy of the product. In order to solve this practical problem, it is necessary to clearly understand the coupling problem of rigid body movement and elastic deformation between the components of the group frame. And the complex dynamics between the large-scale movement of the flexible body and its own deformation. These are current challenges [1]. The structure of the high-speed group machine is very complex. If analyzed, designed, and diagnosed according to conventional methods. Then it may lead to reduced accuracy and increased vibration. The study of the dynamic performance of high-speed group framers has become an important and decisive factor in measuring their working performance. Therefore, how to predict and control the dynamics of the mechanism of the group machine and its performance accurately, in real time and effectively. These have become an important concern for the optimal design and research and development of the assembly machine.

In the optimization of the design of mechanical mechanisms. In order to analyze the dynamic laws of the moving acceleration, shock and vibration of the parts. The researchers built a number of different kinetic models. LIU Yi-fu, SUN Rui-xia [2], Torsional vibration modeling of crankshaft systems using the centralized mass method. This is used to obtain the eigenequations of the system. The two-degree-of-freedom and six-degree-of-freedom equivalent system methods were applied and compared, respectively. From this is the self-resonant frequency and mode of the shaft system and the resonance speed of the low harmonic order is obtained. Zhang Kun, Yang Zhao-jian [3] conducted a series of studies using the centralized mass method to analyze torsional vibrations of rotor bearing systems and monitor them online. Deng Lin [4] used the crankshaft system of an inline four-cylinder engine of a 30T construction vehicle as a research object to establish an ANSYS finite element model of the crankshaft system. Based on the dynamic model, the inherent vibration and forced vibration characteristics of torsional vibration of the shaft system are analyzed. Obtain the main and secondary harmonics in the common speed range and the corresponding engine speed. ZHU Hai-min, CHEN Wei-fang [5], studied the dynamic stability of cam mechanisms based on a centralized parameter model. The influence of elastic vibrations on camshafts and followers is considered in the mechanism dynamics model. This results in a time-varying differential equation for the system. Studies have found that as the speed of the camshaft increases, there will be some unstable areas. And damping has a very important impact on the stability of the system. Ke Fenga, Pietro Borghesani, Wade A. Smith [6], they propose a vibration-based scheme for updating gear wear prediction. Prediction based on models of gearbox dynamics and abrasive wear. Updating of wear constant based on comparing simulated and measured vibration. Scheme allows reliable wear prediction using simple modelling tools. Scheme experimentally validated on run-to-failure dry test with spur gears. Cveticanin [7] studied the dynamic stability of cam mechanisms based on the established centralized parameter model. The influence of elastic vibrations on camshafts and followers is considered in the mechanism dynamics model. This results in a time-varying differential equation for the system. It has found that as the speed of the camshaft increases, there will be some unstable areas. And damping has a very important impact on the stability of the system. Huali Ding, Ahmet Kahraman [8] employs two different dynamic models. A finite elements-based deformable-body model and a simplified discrete model, and a surface wear model are combined to study the interaction between gear surface wear and gear dynamic response. At the end, several sets of simulation results are used to demonstrate the two-way relationship between nonlinear gear dynamics and surface wear. Lassaad et al. [9–11] studied the nonlinear dynamics of cam swing rod mechanisms. The effects of cam roller contact stiffness and bearing support stiffness were taken into account in the dynamic model it constructed. The effect of convex contour error on the kinematic parameters of the follower is also analyzed. However, the error form considered is relatively simple and cannot reflect the actual working conditions of the cam mechanism. Imran S A [12] and Nguyen V [13] build the dynamic model. A vibration analysis of the study object was carried out, and the structure was optimized. Norton R L [14] explored methods to determine the stability and critical speed of elastic mechanisms. The researcher considered the dynamic stability of elastic closed-loop linkage and verified critical speed range of elastic closed-loop linkage system with experiments. Volkan K [15] and Kulesza Z [16] proposed a new finite element method to model the crack of transmission shaft and the model of beam under moving loads. Most of the objects of elastic dynamics analysis are planar mechanisms. There are relatively few spatial mechanisms involved and most of the spatial mechanism analysis is kinematic analysis. However, many achievements have been made after years of development of space mechanism elastic dynamics. But most of current space agency analysis is kinematic analysis. Li Yuning [17] studied the dynamic analysis of crane boom system based on rigid-flexible coupling model. The writer used finite segment method to establish flexible beam model and established the crane boom system dynamics equation of rigid-flexible coupling system based on equivalent finite segment integration method. Then analyzing the luffing starting process of system and studied the dynamic characteristics of lifting point position during luffing starting process. It provides guidance for the related structural design of crane in theory. Zou Xiuqing, Dong Erbao et al. [18] studied the characteristics of beam-type flexible members for SMA flexible torsional actuators. Authors established its discrete analytical model and the finite segment discrete model of SMA flexible torsional actuator. They transform the dynamic problem of flexible mechanism into multi-rigid dynamic problems. So as to realize the analysis of dynamic characteristics of flexible mechanism. M.I. Friswell [19] made a lot of systematic elaboration on the problem of structural dynamics finite element model correction. These provide sufficient theoretical support for subsequent research.

The length of a connecting rod in the cam linkage mechanism is determined according to the kinematic requirements of mechanism. It is generally not suitable to be selected as a variable for the optimal design of mechanism. In the cam linkage mechanism, the cam profile and connecting rod section parameters are selected as design variables to optimize the design. MEI Ju, HUANG Songhe, ZHU Pengfei [20] and others took the cam system as the research object. A spring-mass system is established for a single cam linkage to execute the design requirements of the component as the input signal. The response curve of the cam is obtained under MATLAB/SIMULINK and then the response curve of the cam is used as the input curve of the camshaft. Find the response of the multiple camshafts and find the maximum deformation of the shaft. The institution is then optimized. YUAN S X. [21] has conducted research on the correction of dynamic models of structures at high temperatures. It improves the stability of the components. The convex contour line determines the movement law of the mechanism components. This is the main content of the cam mechanism design work. Flocker [22, 23] proposed a single-dwell and multi-dwell cam follower motion law respectively. And the appropriate adjustment of control parameters in the motion law of follower can meet the requirements of different working conditions. Jiang J K, Iwai YR et al. [24, 25] selected Hermite curve as the motion law of cam followers and studied the vibration problem of cam mechanism. Yang Yuhu; Xie Ran [26] studied a new coaxial indexing mechanism consisting of a conjugated cam and a parallelogram connecting rod mechanism. They established the conjugated cam profile, the radius of curvature of the cam profile, and the meshing equation of the cam and the roller. Then, the main factors affecting the geometry and force transfer performance of the mechanism are analyzed. This study provides a new set of ideas for the mechanical design of indexing mechanisms. Kou Pan and Niu Yanpeng [27, 28] mainly analyzed the influence of the dynamic parameters of valve cam mechanism on the output displacement of the short rocker arm end of valve opening and closing section and cam lift error. At the same time, the influence of dynamic parameters on the residual vibration of valve in the intermittent period is analyzed. The contact model of cam pair is established by introducing the theory of Hertz contact and elastohydrodynamic lubrication. This uses a linear transformation method to eliminate transmission ratio on both sides of rocker arm. They established a set of five-degree-of-freedom dynamic models of valve train unit to obtain the output displacement of each component of valve train. The results show that in the valve opening and closing section, there is an error between the output displacement of short rocker arm end and cam lift; valve train produces serious residual vibration during cam intermittent period. In summary, this paper studies the dynamics of the cam link system of flip mechanism. Based on the MATLAB Genetic Algorithm toolbox [29] and solved using Newmark’s method. Based on the established dynamic model, the dynamic stress, swing force and swing moment of mechanism are analyzed and actuator runs more smoothly and reliably. The dynamic stress, swing force and swing moment of mechanism are analyzed. The result is a smoother and more reliable operation of actuator. In addition, The swing force and swing moment of the mechanism are also selected as the objective functions of optimization model. The node parameters of the motion law of B-spline curve and the section parameters of connecting rod are selected as design variables. The purpose of this is to optimize the design of mechanism. The established optimization model is solved by using genetic algorithm. It is expected that swing force and swing moment of optimized mechanism can be greatly reduced and the vibration of mechanism can be reduced.

## 2 Institutional structural design

### 2.1 Structural design

High-speed assembling machine is a rigid automation equipment with cam mechanism as control system and link mechanism as transmission system. It is suitable for occasions such as aluminum electrolytic capacitors with large production batches and fixed product types. Its working mode first designs the corresponding execution device according to each execution action of assembly process; Secondly, determine the action sequence of each actuator and make a preliminary design of the contour curve of cams based on this; Then, according to the designed actuator and cam mechanism, design space link transmission system; Finally, verify whether the designed mechanism system meets working requirements, and if not, make appropriate corrections to cam link parameters. Based on the in-depth analysis of the process flow of assembly machine, this paper completes the structural design of assembly machine. Kinematics and dynamics of the more complex flip shaping subsystem are analyzed. At the same time, the structure of its flip shaping subsystem is optimized based on genetic algorithm and dynamic model.

**Fig 1:**
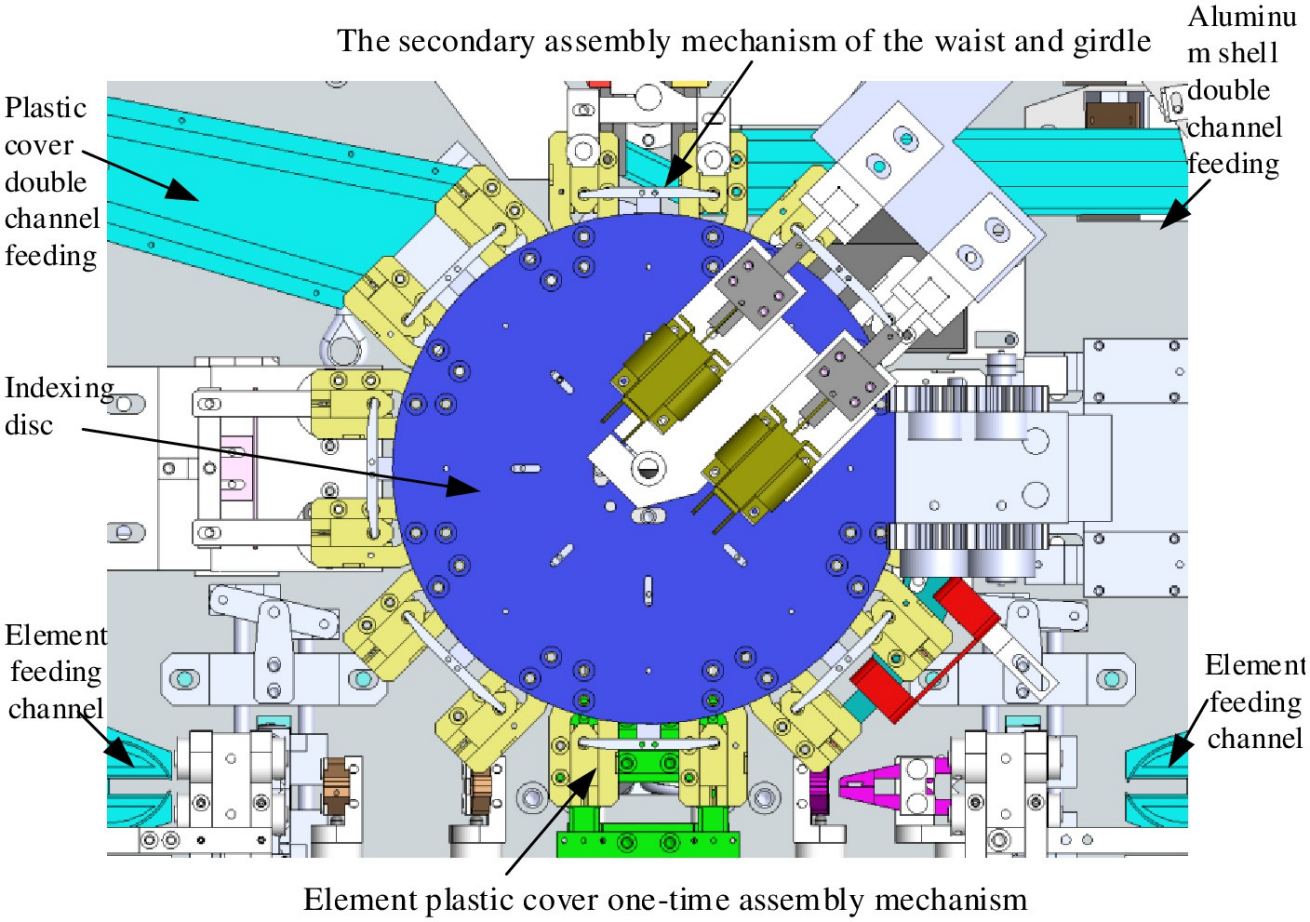
3D model of high-speed assembly machine structure execution.

Fig 1 shows the three-dimensional model of assembling machine actuator designed in the laboratory. Now the process flow of assembly machine is introduced as follows: Pre-process is to send elements to the designated position. A cam in the transfer lead clamp drives the moving clamping part to clamp elements lead and transfer elements; At the same time, the core clamp driven by cam in the transfer core clamp cooperates with lead clamp. An element on the lead clip is connected through core package that clamps the element. And continue to transfer element to shaping module by flipping. At this time, flip transfer in the element transfer is completed. This paper focuses on analyzing the mechanism of a typical flip shaping subsystem.

### 2.2 Flip shaping subsystem

**Fig 2:**
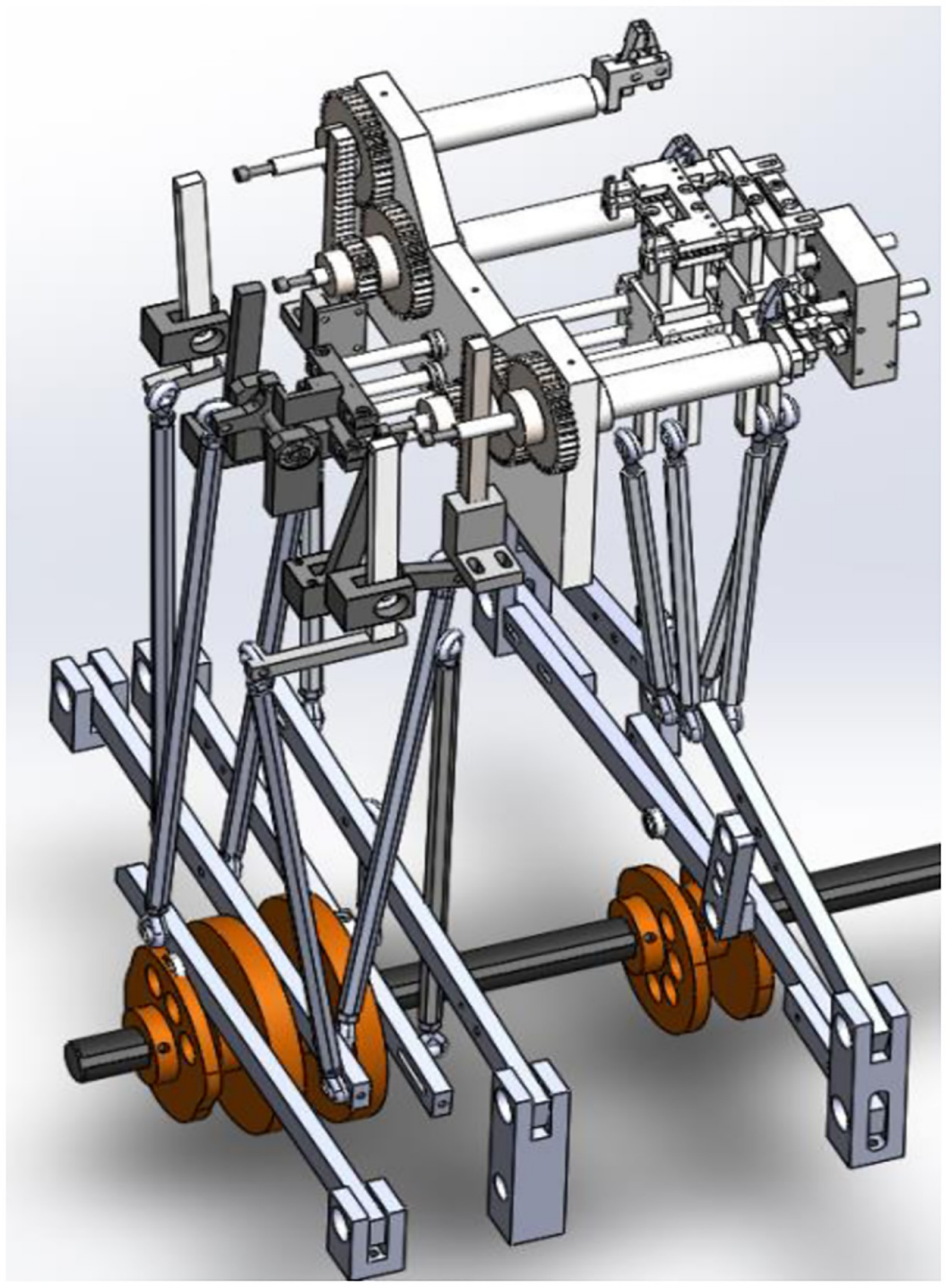
Flip shaping subsystem.

The flip-shaping subsystem mainly completes the flipping of elements and straightens the lead of element. In order to better reflect the structural characteristics of assembly machine. Therefore, transmission control device and the execution device of flip shaping subsystem are put together for analysis. Fig 2 shows three-dimensional model of sub-element flip shaping subsystem of assembly machine. The flip-shaping subsystem consists of four spatial linkages. Motion input and control elements of the flip shaping subsystem are bottom five cams. And its motion transmission components are four space four-bar mechanisms in the middle. Actions such as transferring, flipping, clamping, and shaping are completed by corresponding actuators.

**Fig 3:**
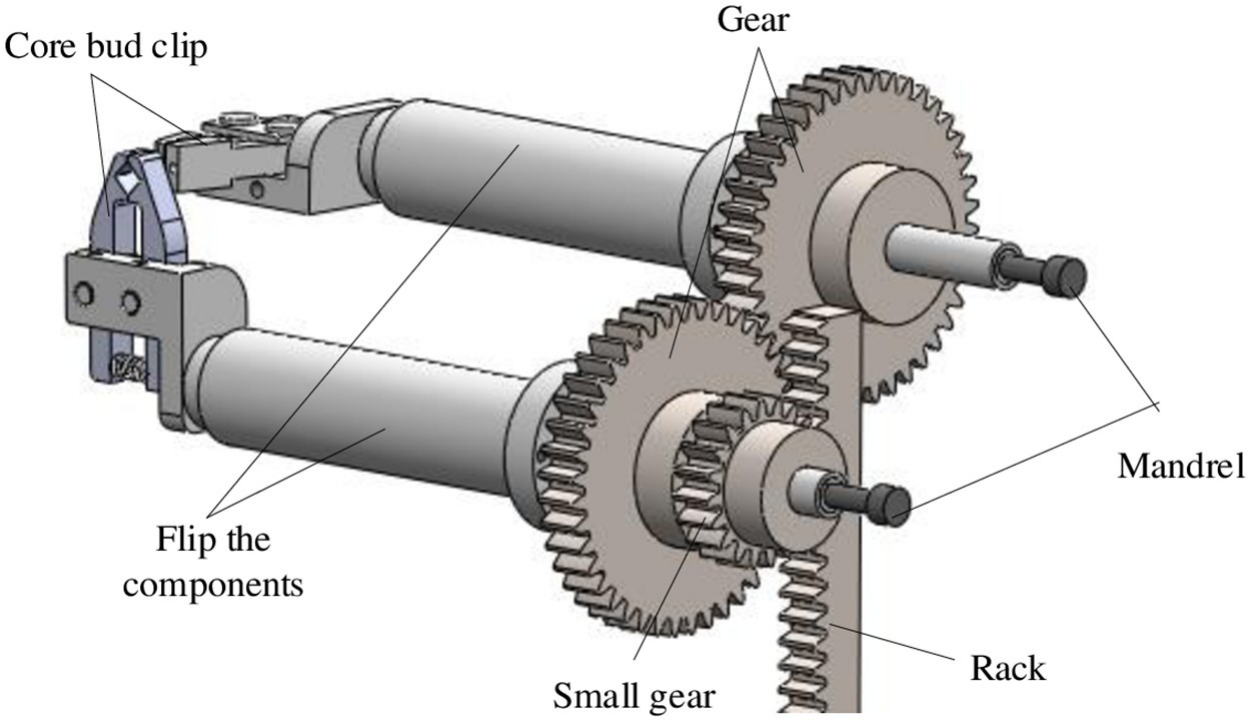
3D model of the actuator of the flip mechanism.

Three-dimensional model of turning mechanism actuator is shown in Fig 3. The up and down movement of rack is converted into the opposite rotation of flipping assembly through a rack and pinion transmission. And the element core package is clamped by core bud clip to complete the flipping action of element. Opening and closing of the core bud clip is controlled by the rack and pinion transmission in the figure.

## 3 Construction of kinematic equations and result analysis of flip shaping mechanism

### 3.1 Construction of the kinematic equation of flip-shaping mechanism

The cam link mechanism is used as control transmission part of assembling machine to make the structure of assembling machine more compact. But there is also an obvious disadvantage. That is, the power balance of mechanism is poor. Linkage transmission produces a large inertial excitation. This will make mechanical system produce larger vibration and noise. When the vibration of mechanism is large, overturning and shaping mechanism will not be able to accurately complete overturning and shaping action of element. Two pins of the element may be stuck between two clips closed on the top and bottom of assembly machine. So that machine can not work properly. For this reason, the kinematics analysis of overturning shaping mechanism in the overturning shaping subsystem of erecting machine is carried out. The purpose of this is to explore why structure vibrates.

**Fig 4:**
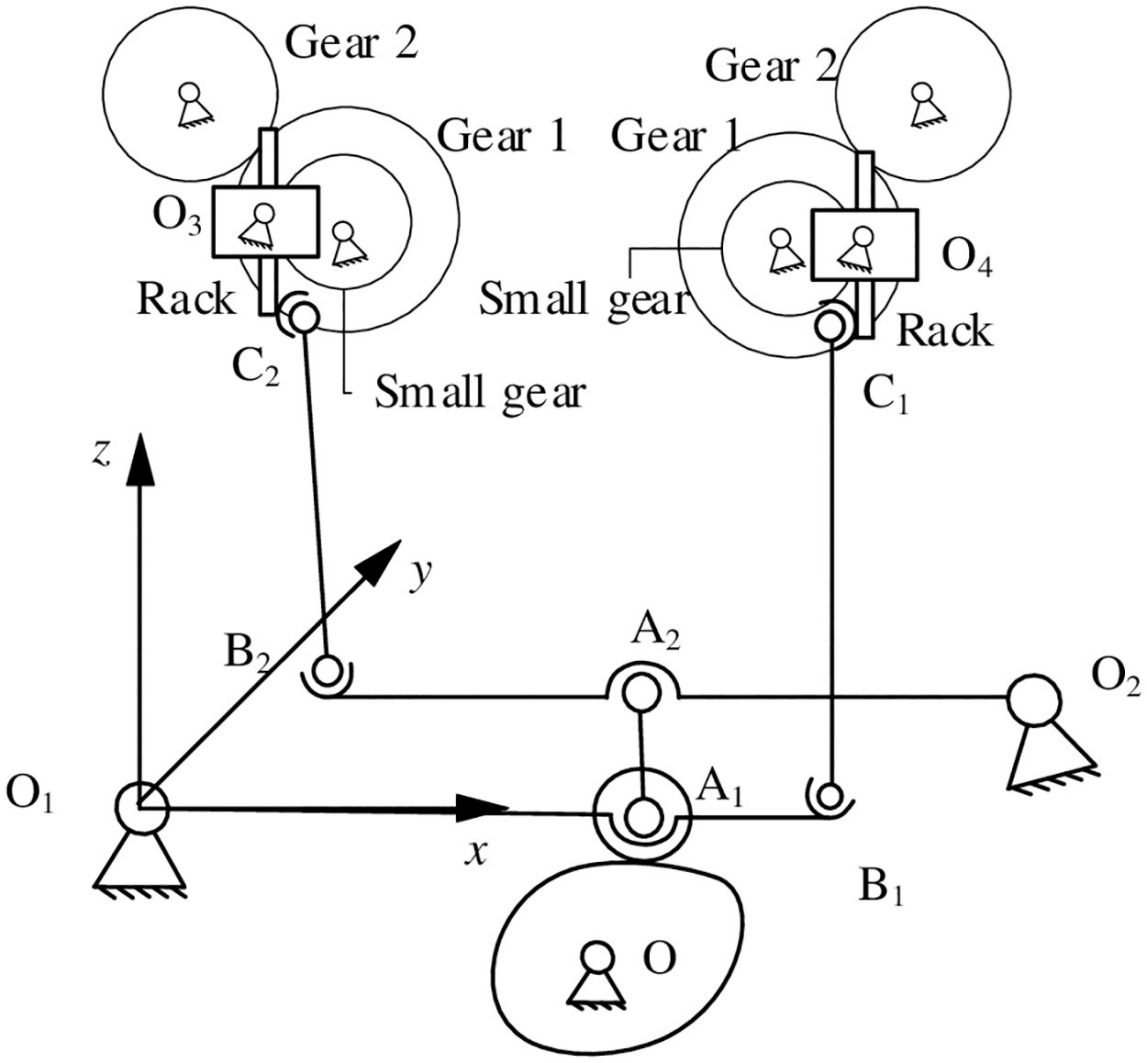
The schematic diagram of flipping mechanism.

Fig 4 shows the schematic diagram of flip-shaping mechanism and establishes a coordinate system. The overturning mechanism is mainly composed of a cam pendulum mechanism, two crank-slider mechanisms and a rack and pinion overturning assembly mechanism. A cam pendulum mechanism is motion input part of the turning system; Space crank-slider mechanism O_1_B_1_C_1_ and O_2_B_2_C_2_ convert motion input by cams into the up and down movement of racks. It is motion transmission part of the turning mechanism system; A rack and pinion overturning assembly is the actuator of overturning mechanism. It completes the flipping action of capacitor.

#### 3.1.1 Construction of the kinematic equation of flip mechanism

Set coordinate system O_1_-xyz as shown in Fig 4 in the flip shaping mechanism. In the mechanism motion engineering, the length of connecting rod B_1_C_1_ in the crank-slider mechanism O_1_B_1_C_1_ remains unchanged. This can obtain the kinematic equation of space crank-slider mechanism O_1_B_1_C_1_ as shown in the following formula (1):

$${\left(l_{{\text{O}}_{1}{\text{B}}_{1}}\;\text{cos}\;\theta_{1}-d_{C_{1}x}\right)}^{2}+d_{C_{1}y}{}^{2}+{\left(l_{{\text{O}}_{1}{\text{B}}_{1}}\;\text{sin}\;\theta_{1}-s_{C_{1}}\right)}^{2}=l_{{\text{B}}_{1}{\text{C}}_{1}}^{2}$$ (1)

In the same way, a kinematic equation of space crank-slider mechanism O_2_B_2_C_2_ can be obtained as shown in formula (2):

$${\left(l_{{\text{O}}_{2}{\text{B}}_{2}}\;\text{cos}\;\theta_{2}+d_{O_{2}x}-d_{C_{2}x}\right)}^{2}+d_{C_{2}y}{}^{2}+{\left(l_{{\text{O}}_{2}{\text{B}}_{2}}\;\text{sin}\;\theta_{2}+d_{O_{2}z}-s_{C_{2}}\right)}^{2}=l_{{\text{B}}_{2}{\text{C}}_{2}}^{2}$$ (2)

In the formula, *θ*_1_, *θ*_2_ are the swing angles of pendulum rods O_1_B_1_ and O_2_B_2_, respectively. $l_{{\text{O}}_{1}{\text{B}}_{1}}$, $l_{{\text{O}}_{2}{\text{B}}_{2}}$ represent the length of O_1_B_1_ and O_2_B_2_, respectively. $d_{{\text{O}}_{2}x}$, $d_{{\text{O}}_{2}z}$ represent the projection of coordinates of O_2_ point on the *x*-axis and *y*-axis. $d_{C_{1}x}$, $d_{C_{1}y}$ are the projections of point C_1_ on the *x*-axis and *y*-axis. $s_{C_{1}}$ represents the displacement of point C_1_ in the *z*-axis direction. $d_{C_{2}x}$, $d_{C_{2}y}$ represent the projection of point C_2_ on the *x* and *y* axes. $s_{C_{2}}$ indicates the displacement of point C_2_ in the *z*-axis direction.

The plane four-bar mechanism is composed of pendulum rods O_1_A_1_, A_1_A_2_ and O_2_A_2_. According to the fixed length constraint of pendulum rod A_1_A_2_, the motion equation of mechanism can be obtained as shown in formula (3):

$${\left(l_{{\text{O}}_{1}{\text{A}}_{1}}\;\text{cos}\;\theta_{1}-l_{{\text{O}}_{2}{\text{A}}_{2}}\;\text{cos}\;\theta_{2}-d_{{\text{O}}_{2}x}\right)}^{2}+{\left(l_{{\text{O}}_{1}{\text{A}}_{1}}\;\text{sin}\;\theta_{1}-l_{{\text{O}}_{2}{\text{A}}_{2}}\;\text{sin}\;\theta_{2}-d_{{\text{O}}_{2}z}\right)}^{2}=l_{{\text{A}}_{1}{\text{A}}_{2}}^{2}$$ (3)

In the formula, $l_{{\text{O}}_{1}{\text{A}}_{1}}$ $l_{{\text{O}}_{2}{\text{A}}_{2}}$ represent the length of connecting rod O_1_A_1_ and O_2_A_2_. $l_{{\text{A}}_{1}{\text{A}}_{2}}$ is the length of connecting rod A_1_A_2_.

The input motion of known cam combines above three equations. This can calculate the kinematic relationship of sliders C_1_ and C_2_.

#### 3.1.2 Results analysis and discussion

**Fig 5:**
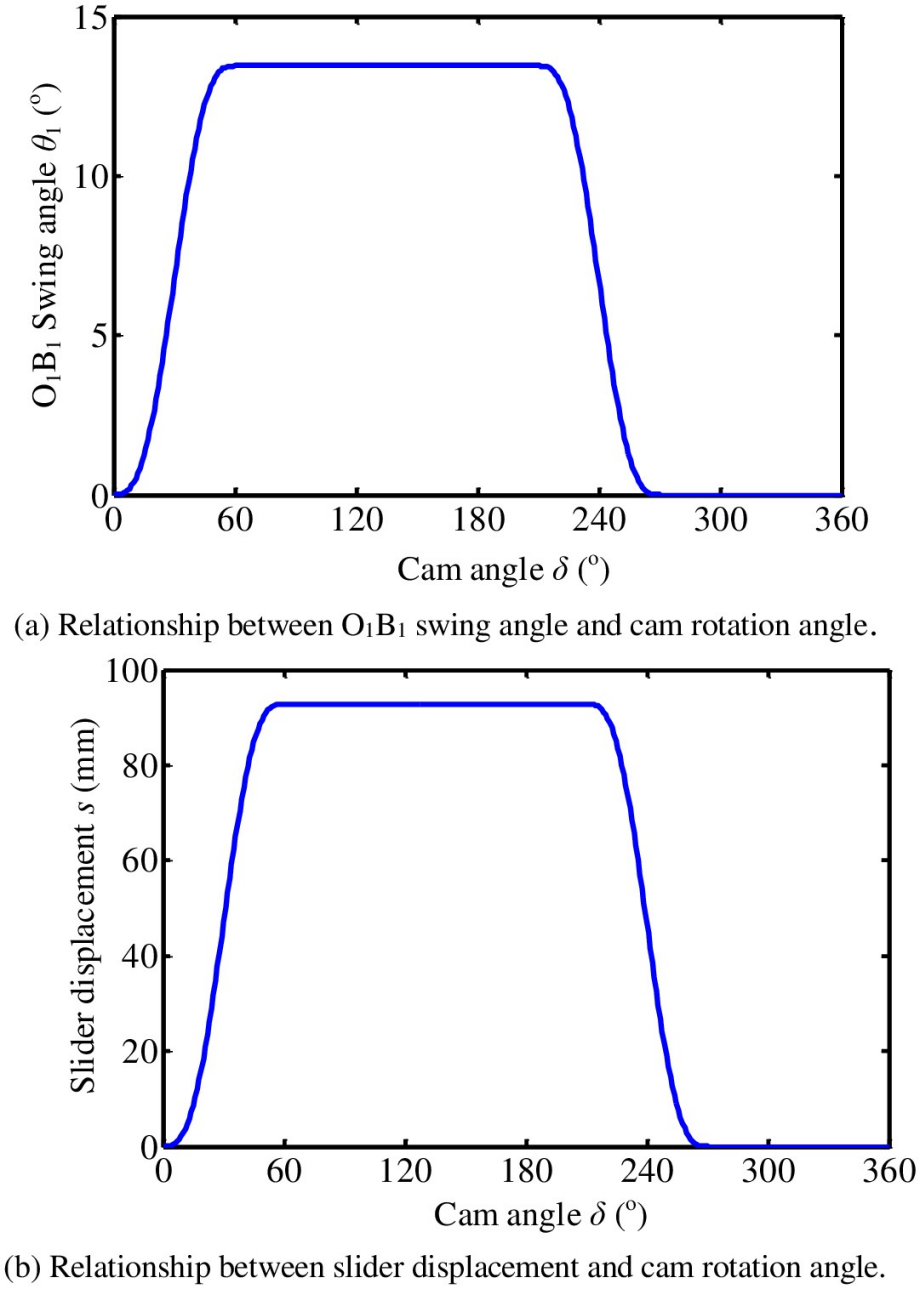
Motion law of the cam of the flip mechanism. (a) Relationship between O_1_B_1_ swing angle and cam rotation angle. (b) Relationship between slider displacement and cam rotation angle.

It can be seen from the displacement curve of a cam motion law of flip-shaping mechanism in Fig 5. The velocity and acceleration curves are continuous. This characterizes it with good kinetic properties. Therefore, when designing the cam, cam profile curve is designed according to the law of cycloid motion.

## 4 Construction and characteristic analysis of the dynamic model of turning mechanism

### 4.1 Dynamic equation of the link subsystem of flip mechanism

**Fig 6:**
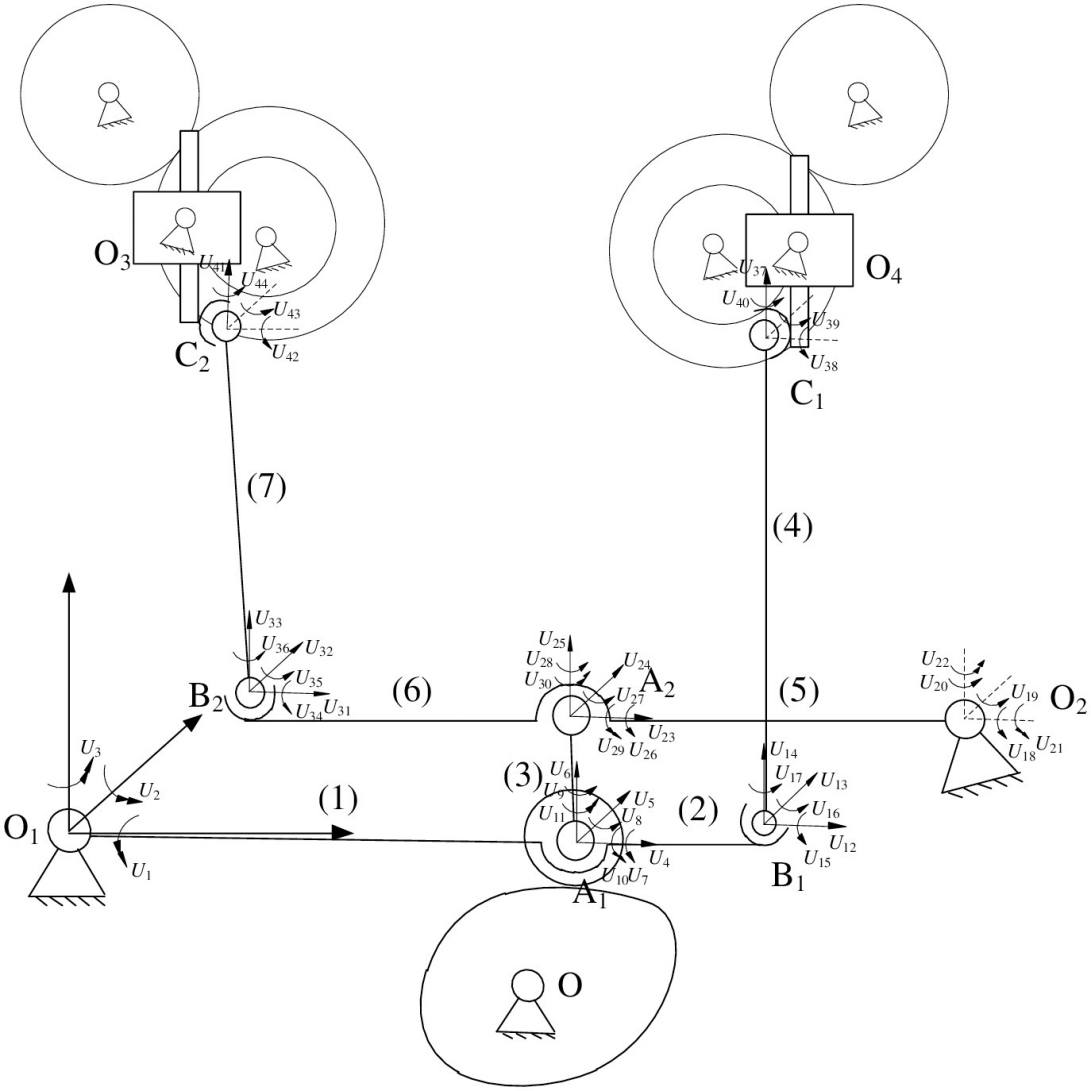
Unit division of the link subsystem of flip mechanism.

The dynamic equation of linkage system of flip mechanism can be established by finite element method. The unit division of linkage mechanism is shown in Fig 6. In order to eliminate the degrees of freedom of linkage subsystem of mechanism. Rotation pair O_1_ is assumed to be the fixed end and only three curvature coordinates are set there. A rack and pinion and other components in the mechanism have high rigidity and are treated as rigid bodies. The linkage subsystem of flip mechanism is divided into 7 units and 44 generalized coordinates are set. According to the dynamic equation of beam element derived earlier. The dynamic equation of unit *i* (*i* = 1, 2, 3,…,7) in the connecting rod subsystem is shown in Eq (4):

$$M_{ei}{\ddot{U}}_{i}+C_{ei}{\dot{U}}_{i}+K_{ei}U_{i}=P_{ei}+Q_{ei}+F_{ei}$$ (4)

In the formula, ***P***_*ei*_ is inertial force on the element. ***Q***_*ei*_ is the force of adjacent element on the element *i*. ***F***_*ei*_ is generalized external force on the element *i*. And ***U***_*i*_ is the generalized coordinate array of element *i*.

The relationship between elastic coordinate *Ui* of link subsystem unit *i* and generalized coordinate U* of the link subsystem can be expressed by coordinate coordination matrix ***B***_***i***_:

$$U_{i}=B_{i}U^{*}$$ (5)

In the formula, U* = [*U1*, *U2*, *U3…U42*, *U43*, *U44*]^T^ is the generalized coordinate of linkage subsystem. The elements in the coordinate coordination matrix ***B***_*i*_ are either 0 or 1. The position of matrix element in ***B***_*i*_ represents the corresponding position of generalized coordinate *j* in the unit *i* in the generalized coordinate of system.

Substitute Eq (5) into the dynamic Eq (4) of mechanism unit. Multiplying both sides of the equation by $B_{i}^{T}$ yields element dynamics equation represented by the generalized coordinates of connecting rod subsystem:

$$M_{i}{\ddot{U}}^{*}+C_{i}{\dot{U}}^{*}+K_{i}U^{*}=P_{i}+Q_{i}+F_{i}$$ (6)

In the formula, $M_{i}=B_{i}^{T}M_{ei}B_{i}$, $C_{i}=B_{i}^{T}C_{ei}B_{i}$, $K_{i}=B_{i}^{T}K_{ei}B_{i}$, $P_{i}=B_{i}^{T}P_{ei}$, $Q_{i}=B_{i}^{T}Q_{ei}$, $F_{i}=B_{i}^{T}F_{ei}$.

The unit dynamics Eq (6) is assembled to obtain the dynamics equation of connecting rod subsystem, as shown in Eq (7):

$$M{\ddot{U}}^{*}+C{\dot{U}}^{*}+KU^{*}=P+F$$ (7)

In the formula, $M=\sum_{i=1}^{7}M_{i}$, $C=\sum_{i=1}^{7}C_{i}$, $K=\sum_{i=1}^{7}K_{i}$, $P=\sum_{i=1}^{7}P_{i}$, $F=\sum_{i=1}^{7}F_{i}$.

In the element kinetic Eq (7). ***Q***_*i*_ is the internal force of connecting rod subsystem. During the process of assembling unit equations into system equations, ***Q***_*i*_ cancels each other out. Therefore, this term is not included in the system dynamics Eq (7). ***F*** is the generalized external force array on the connecting rod subsystem. For the link subsystem of flip mechanism, ***F*** mainly includes the force of racks and pinions on the link subsystem.

Both ends of space links B_1_C_1_ and B_2_C_2_ in the turning mechanism are spherical pairs. The connecting rod has local degrees of freedom to rotate around itself. Therefore, connecting rods B_1_C_1_ and B_2_C_2_ do not have torsional deformation. The elastic corner coordinates of unit 4 and unit 7 are not independent of each other. This is discussed using three elastic corner coordinates at node B_1_ as an example. The ***u***B_1_ = [*u*_15_*u*_16_*u*_17_]^*T*^ is the elastic corner coordinate of node B in the element coordinate system. ***U***_B1_ = [*U*_15_*U*_16_*U*_17_]^*T*^ is elastic corner coordinate expressed in the global coordinate system. Let ***T*** be coordinate transformation matrix between link BC unit coordinate system and global coordinate system, then:

$$u_{\text{B}1}=T^{T}U_{\text{B}1}$$

Connecting rod B_1_C_1_ does not produce torsional deformation. Therefore, there are:

$$u_{17}=r_{31}U_{15}+r_{32}U_{16}+r_{33}U_{17}=0$$ (8)

In the formula, *r11*, *r21*, and *r31* are the first column elements in the coordinate transformation matrix ***T***.

Select *U*_*15*_ and *U*_16_ as the independent coordinates of elastic corner at node **B**. Then there is the following conversion relationship:

$$\left[\begin{array}{c} U_{15}\\ U_{16}\\ U_{17} \end{array}\right]=\left[\begin{array}{cc} 1 & 0\\ 0 & 1\\ -r_{31}/r_{33} & -r_{32}/r_{33} \end{array}\right]\left[\begin{array}{c} U_{15}\\ U_{16} \end{array}\right]=B_{r}\left[\begin{array}{c} U_{15}\\ U_{16} \end{array}\right]$$ (9)

Select ***U*** = [*U*1, *U*2…*U*15, *U*16, *U*18…*U*33, *U*34, *U*35, *U*37, *U*38, *U*39, *U*41, *U*42, *U*43]^*T*^ as the independent generalized coordinates of system. Then the transformation relationship between independent generalized coordinate ***U*** of connecting rod subsystem and generalized coordinate U* of the system is shown in formula (10):

$$U^{*}=BU$$ (10)

In the formula, ***B*** is coordinate transformation matrix and B = diag (***I***_14×14_, ***B***_*r*1_, ***I***_16×16_, ***B***_*r*2_, ***U***_37_, ***B***_*r*1_, ***U***_41_, ***B***_*r*2_). Substitute Eq (10) into the dynamic Eq (7) of connecting rod subsystem and left-multiply ***B***^*T*^ to get:

$$M_{s}\ddot{U}+C_{s}\dot{U}+K_{s}U=P_{s}+F_{s}$$ (11)

In the formula, ***M***_*s*_ = ***B***^*T*^***MB***, ***C***_*s*_ = ***B***^*T*^***CB***, ***K***_*s*_ = ***B***^*T*^***KB***, ***P***_*s*_ = ***B***^*T*^***P***, ***F***_*s*_ = ***B***^*T*^***F***.

Racks and pinions flipping components in the flipping mechanism are assumed to be rigid bodies. This should be described by the rigid body dynamics equations. Select a rack as equivalent component. Then the equivalent mass of this part is:

$$m_{e}=m_{1}+\frac{J_{1}}{r_{1}^{2}}+\frac{J_{2}i^{2}}{r_{1}^{2}}$$ (12)

In the formula, *m*_*e*_ represents the equivalent mass of the part. *m*_1_ is rack mass. *J*_1_ is the moment of inertia of lower clip flip assembly. *J*_2_ is the moment of inertia of the upper clip flip assembly. *i* is the transmission ratio of two gears. *r*_1_ is radius of the indexing circle of pinion.

Rigid body dynamics equation of rack and pinion turning assembly is shown in Eq (13):

$$\left\{\begin{array}{l} m_{e}{\ddot{U}}_{37}=F_{1}-m_{e}s_{\text{C}1}\\ m_{e}{\ddot{U}}_{41}=F_{2}-m_{e}s_{\text{C}2} \end{array}\right.$$ (13)

In the formula, *F*_1_ and *F*_2_ are the interaction forces between rack and connecting rods B_1_C_1_ and B_2_C_2_, respectively.

Superimposing Eq (13) into dynamic Eq (11) yields the dynamic equations of connecting rod subsystem:

$$M_{L}\ddot{U}+C_{L}\dot{U}+K_{L}U=P_{L}+F_{L}$$ (14)

Eq (14) is dynamic equation that includes the rigid body motion of the rack and pinion flip assembly. The main difference compared to Eq (11) is that mass matrix ML in Eq (14) includes the inertia of the flipped component. And the generalized external force array ***F***_*L*_ only includes contact force at the cam roller. The forces between rack and link subsystems are cancelled out during the superposition of equations.

**(1) Damping of connecting rod system**. Damping is an important factor that must be considered in the dynamic analysis of mechanical systems. Damping dissipates the energy of system and gradually dampens the vibration response of the mechanical system. There are many reasons for the damping of mechanical systems. It is not only closely related to the material properties of mechanism components. The actual working conditions of the mechanical system (friction, lubrication, etc.) also have a non-negligible impact on the damping of the system. In practical applications, the damping is usually assumed to be viscous damping proportional to the velocity. In the specific calculation, damping matrix ***C*** of the mechanical system is generally taken as the sum of the ratios of mass matrix ***M*** and stiffness matrix ***K***. As shown in formula (15):

C=αM+βK (15)

*α* and *β* in the formula are Rayleigh damping proportional coefficients. Calculated according to following formula

$$\alpha =\frac{2\left(\xi_{i}\omega_{j}-\xi_{j}\omega_{i}\right)}{\left(\omega_{j}+\omega_{i}\right)\left(\omega_{j}-\omega_{i}\right)}\omega_{i}\omega_{j},\beta =\frac{2\left(\xi_{j}\omega_{j}-\xi_{i}\omega_{i}\right)}{\left(\omega_{j}+\omega_{i}\right)\left(\omega_{j}-\omega_{i}\right)}$$

ω_i_ and ω_j_ in the above formula are any two-order natural frequencies of the mechanical system. ξi and ξj are corresponding damping ratios. In engineering, the first two orders of frequency and damping ratio are usually used for calculation. Damping scale factor is obtained experimentally.

## 5 Dynamics model of cam roller subsystem of flip mechanism

### 5.1 Cam roller subsystem model

**Fig 7:**
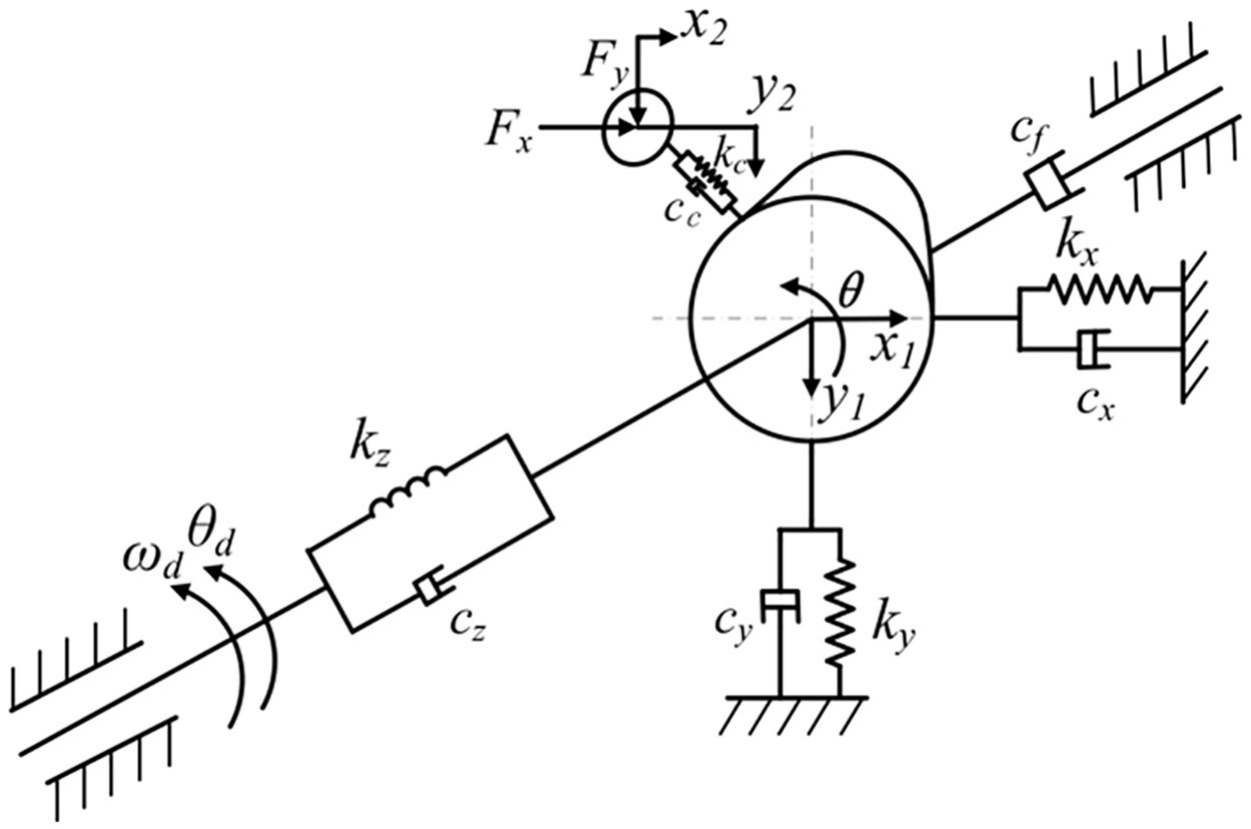
Diagram of dynamic model of cam roller subsystem.

**Fig 8:**
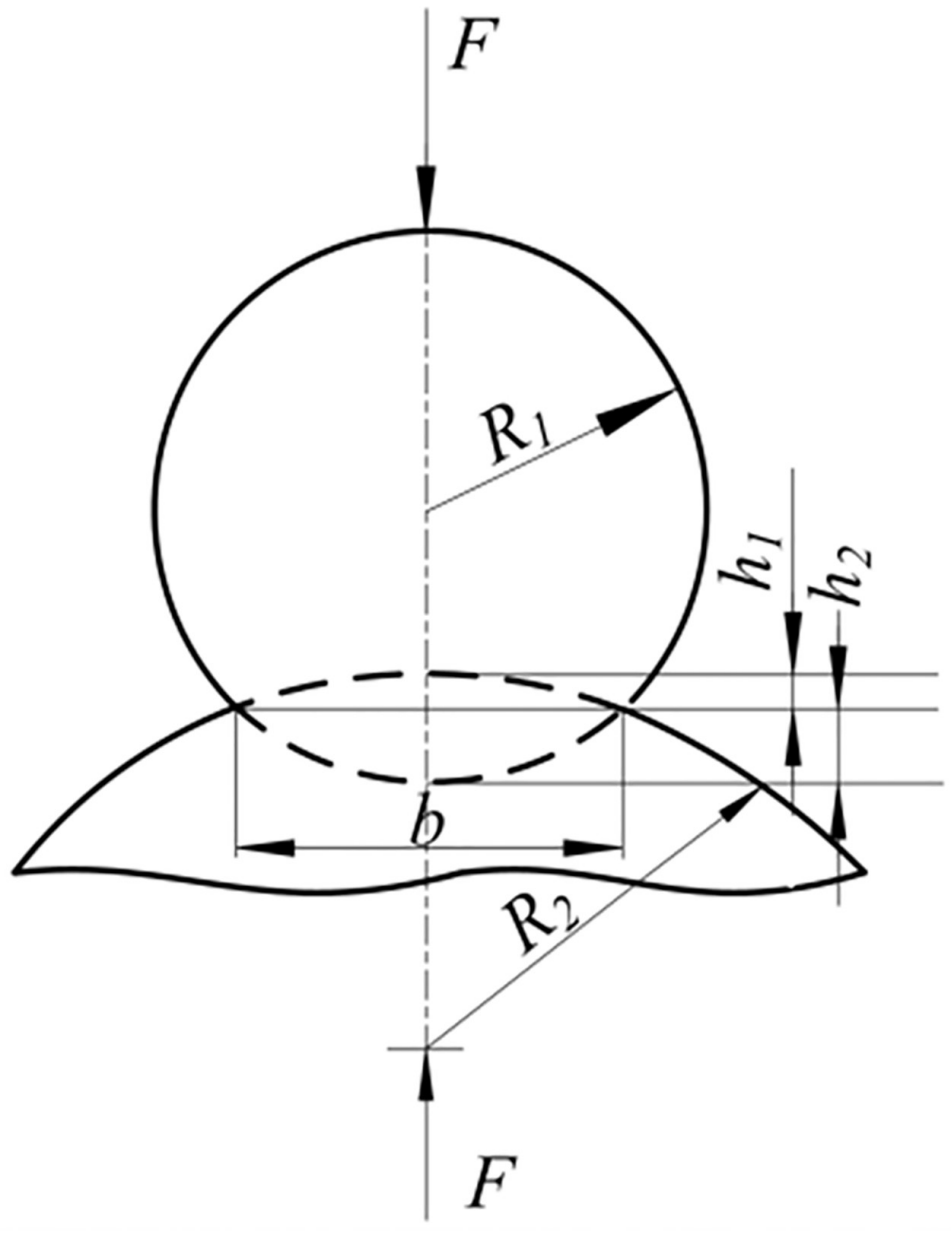
Sketch map of cam roller contact model.

The dynamic model of cam roller subsystem of overturning mechanism will be established by lumped parameter method. Fig 7 shows a simplified diagram of the dynamics model of this subsystem. Cams and camshafts in the turning mechanism are simplified to concentrated mass *m*_1_ and the concentrated moment of inertia *Jz* in Fig 8. Stiffness *k*_*z*_ in the model represents the torsional stiffness of camshaft. *k*_*x*_, *k*_*y*_ represent the bending stiffness of camshaft in the x-direction and the *y*-direction. Rollers in the cam roller subsystem are simplified to concentrated mass *m*_2_. In the figure *k*_*c*_ is contact stiffness between the cam and roller.

Referring to Fig 7, the dynamic equation of cam roller subsystem is shown in Eq (16):

$$\left\{\begin{array}{l} m_{1}{\ddot{x}}_{1}=-k_{x}x_{1}-c_{x}{\dot{x}}_{1}+k_{cx}(x_{2}-x_{1})+c_{cx}({\dot{x}}_{2}-{\dot{x}}_{1})\\ m_{1}{\ddot{y}}_{1}=-k_{y}y_{1}-c_{y}{\dot{y}}_{1}+k_{cy}(y_{2}-y_{1})+c_{cy}({\dot{y}}_{2}-{\dot{y}}_{1})\\ J_{z}\ddot{\theta }=k_{z}(\theta_{d}-\theta )+c_{z}({\dot{\theta }}_{d}-\dot{\theta })-c_{f}\dot{\theta }-M_{c}\\ m_{2}{\ddot{x}}_{2}=-k_{cx}(x_{2}-x_{1})-c_{cx}({\dot{x}}_{2}-{\dot{x}}_{1})+F_{x}\\ m_{2}{\ddot{y}}_{2}=-k_{cy}(y_{2}-y_{1})-c_{cy}({\dot{y}}_{2}-{\dot{y}}_{1})+F_{y} \end{array}\right.$$ (16)

In the formula, *c*_*z*_, *c*_*x*_, *c*_*y*_ and *c*_*c*_ are damping corresponding to torsional stiffness, bending stiffness and cam roller contact stiffness of camshaft. *θ*_*d*_ is the input angle of camshaft and *θ* is the angular position of cam. *x*_1_, *y*_1_ and *x*_2_, *y*_2_ are the elastic displacements of cam and roller, respectively. *c*_*f*_ is friction damping at the camshaft bearing support. *F*_*x*_ and *F*_*y*_ are interaction forces between roller and pendulum rod OA. *M*_*c*_ represents the torque of rollers to the camshaft.

Arrange Eq (16) into a matrix form as shown in Eq (17):

$$M_{T}{\ddot{x}}_{1}+C_{T}{\dot{x}}_{1}+K_{T}x_{1}=F_{T}$$ (17)

**(1) Cam roller contact stiffness and damping**. The contact between cam rollers can be simplified as cylindrical contact model shown in Fig 8. The contact stiffness between cam rollers can be calculated by formula (18):

$$k_{c}=\frac{F}{h_{1}+h_{2}}$$ (18)

In the formula, *F* is the contact pressure between cam rollers. And *h*_1_ and *h*_2_ respectively represent the contact deformation depth of cam rollers.

Assume that axial contact length between cam rollers is *B*. Then the contact deformation width of the cam roller can be obtained according to Hertz contact formula:

$$b=4\sqrt{\frac{F}{\pi B}\frac{\frac{1-\mu_{1}^{2}}{E_{1}}+\frac{1-\mu_{2}^{2}}{E_{2}}}{\frac{1}{R_{1}}\pm \frac{1}{R_{2}}}}$$ (19)

In the formula, *μ*_1_ and *μ*_2_ are the Poiss’s ratios of the cam and roller materials. *E*_1_ and *E*_2_ are the elastic moduli of cam and roller materials. *R*_1_ and *R*_2_ are the radii of curvature of the cam and roller at the contact point.

According to geometric relationship shown in Fig 8, the deformation depth of a cam roller in the contact area is shown in Eq (20):

$$\left\{\begin{array}{l} h_{1}=R_{1}-\sqrt{R_{1}^{2}-b^{2}/4}\\ h_{2}=R_{2}-\sqrt{R_{2}^{2}-b^{2}/4} \end{array}\right.$$ (20)

Simultaneously (18), (19) and (20) can calculate the contact stiffness of cam roller. Since the radius of curvature of the cam at the contact point varies with cam angle position within a certain range. Therefore, the contact stiffness of cam rollers also has a range of variation.

The contact damping between cam rollers is calculated according to Eq (21):

$$c=2\xi \sqrt{\frac{m_{1}\cdot m_{2}}{m_{1}+m_{2}}k_{12}}$$ (21)

where *m*_1_ and *m*_2_ are corresponding mass parameters, *k*_12_ is contact stiffness and *ζ* is damping ratio.

## 6 Solution of the dynamic model of the flipping mechanism

### 6.1 Dynamic equation solving method

Eqs (14) and (16) are system dynamics equations of the flipping mechanism. Mass matrix ***M*** and stiffness matrix ***K*** in the equation are constantly changing with the movement of the mechanism. This kinetic equation is a system of differential equations with variable coefficients and its exact solution is difficult to obtain. For such problems, time-discrete method is often used in engineering to solve them. That is to say, one motion period *T* of the mechanism is discretized into several time units Δ*t*. The coefficient matrix of the mechanism dynamics equation can be regarded as constant in each time unit. At present, the commonly used methods for solving the dynamic equations of mechanisms mainly include mode shape superposition method and direct integration method.

Newmark method in the direct integration method is used in this paper. It is based on Lagrange’s mean value theorem and makes approximate assumptions about velocity vector at time *t*+Δ*t*. When the parameters satisfy certain conditions, Newmark method is unconditionally stable. The stability, calculation accuracy and speed of system dynamics equations are comprehensively considered. The Newmark method is used in this paper and specific calculation process is as follows:

1. The correlation coefficient matrix (***M*, *C*, *K***) of the system dynamics equation and the initial state vector of the system ***U***_0_, ${\dot{U}}_{0}$ and ***Ü***_0_ are obtained by analyzing the flipping mechanism.
2. Select the appropriate time step Δ*t* and solution parameters *μ*_1_, *μ*_2_. And calculate the following solution parameters: *a*_0_ = 1/(*μ*_2_Δ*t*^2^), *a*_1_ = *μ*_1_/(*μ*_2_Δ*t*), *a*_2_ = 1/(*μ*_2_Δ*t*), *a*_3_ = 1/(2*μ*_2_)-1, *a*_4_ = *μ*_1_/*μ*_2_−1, *a*_5_ = *μ*_1_Δ*t*/(2*μ*_2_)-Δ*t*, *a*_6_ = Δ*t*(1-*μ*_1_), *a*_7_ = *μ*_1_Δ*t*.
3. Calculate the effective stiffness matrix of the system: $\bar{K}=K+a_{0}M+a_{1}C$.
4. Calculate payload and elastic motion parameters of the system at time *t*+Δ*t*:

${\bar{F}}_{t+\Delta t}=F_{t+\Delta t}+M\left(a_{0}U_{t}+a_{2}{\dot{U}}_{t}+a_{3}{\ddot{U}}_{t}\right)+C\left(a_{1}U_{t}+a_{4}{\dot{U}}_{t}+a_{5}{\ddot{U}}_{t}\right)$

$U_{t+\Delta t}={\bar{K}}^{-1}{\bar{F}}_{t+\Delta t},\;{\ddot{U}}_{t+\Delta t}=a_{0}\left(U_{t+\Delta t}-U_{t}\right)-a_{2}{\dot{U}}_{t}-a_{3}{\ddot{U}}_{t},$

${\dot{U}}_{t+\Delta t}={\dot{U}}_{t}+a_{6}{\ddot{U}}_{t}+a_{7}{\ddot{U}}_{t+\Delta t}$

### 6.2 Influence of camshaft speed on the elastic dynamic characteristics of mechanism

**Fig 9:**
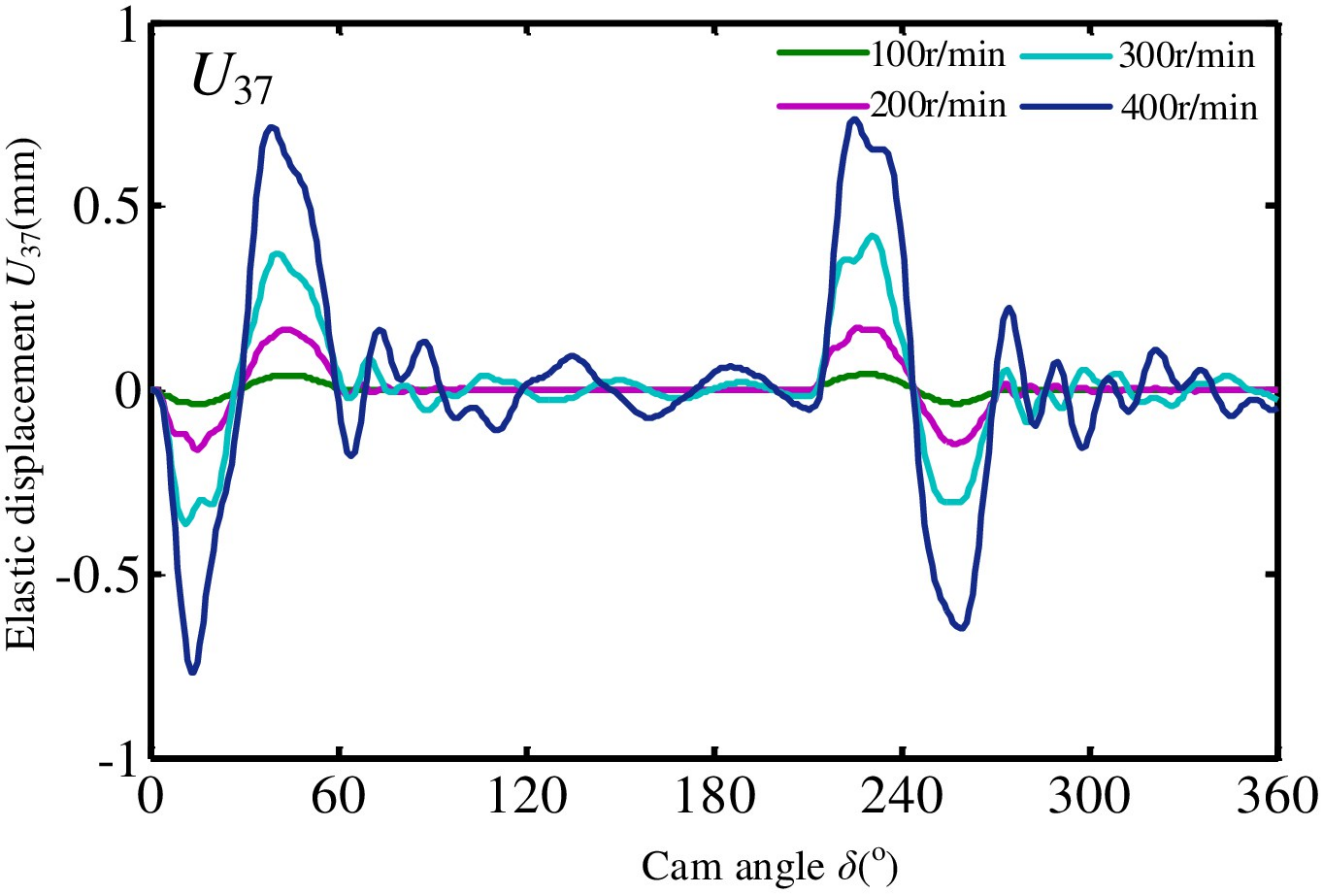
Change curve of elastic displacement of connecting rod of flip mechanism.

According to the dynamic equation of flipping mechanism system established above and Newmark method. It can be solved by MATLAB programming. The following takes the elastic displacement of hinge point C_1_ between the rack and the connecting rod of flip mechanism in the z-axis direction as an example to analyze the influence of camshaft speed on the elastic dynamic characteristics of the mechanism. It can be seen from Fig 9 that camshaft speed has an important influence on the dynamic characteristics of shear mechanism. With the increase of camshaft speed, the vibration amplitude of the elastic displacement of the node increases nonlinearly. The mechanism components not only have large vibrations in the movement phase, but also produce large residual vibrations in the rest phase of the mechanism.

### 6.3 Analysis of swing force and swing moment of the mechanism

The magnitude and direction of swing force (swing moment) of mechanism change periodically. It refers to resultant force (resultant moment) transmitted to frame by the mechanism due to inertial action. And swing force and swing moment transmitted by components and kinematic pairs to frame also change periodically. It is the main cause of vibration of the mechanism on the base. The dynamic equation of linkage subsystem is composed of dynamic equations of each unit of the mechanism. When elastic displacement ***U*** and elastic acceleration ***Ü*** of each node are obtained from the system equation. Transformation is performed according to the inverse process of coordinate transformation process described above. Elastic displacement ***U***_e_ and elastic acceleration ***Ü***_*e*_ of each element can be obtained. Substituting this into the element dynamics equation yields:

$$Q_{ei}=M_{ei}{\ddot{U}}_{i}+C_{ei}{\dot{U}}_{i}+K_{ei}U_{i}-P_{ei}-F_{ei}$$ (22)

In the formula, ***Q***_*ei*_ is the nodal external force of the element i. When the position of the element node coincides with the position of the kinematic pair of the mechanism, ***Q***_*ei*_ is reaction force of the kinematic pair.

Since swing force (swing moment) is resultant force (result moment) transmitted by the mechanism to the frame. Therefore, the swing force (swing moment) is closely related to the reaction force of the kinematic pair at the connecting frame pairs O, D, E and H in the shearing mechanism. The expression of the swing force of the mechanism is shown in formula (23):

$$F_{x}=\sum_{k=1}^{4}F_{kx},\;\;\;F_{y}=\sum_{k=1}^{4}F_{ky},\;\;\;F_{z}=\sum_{k=1}^{4}F_{kz}$$ (23)

In the formula, *F*_*x*_, *F*_y_ and Fz are the components of swing force on the three coordinate axes. And *F*_*kx*_, *F*_*ky*_, *F*_*kz*_ represent three coordinate components of the motion pair reaction force of the connecting frame pair *k*.

The calculation of the swing moment of a mechanism is related to the selection of the reference point. In the shearing mechanism, select the motion pair O_1_ as reference point. Then swinging moment of the mechanism is shown in formula (24):

$$\left\{\begin{array}{l} M_{x}=\sum_{k=1}^{4}\left(M_{kx}+F_{ky}d_{kz}+F_{kz}d_{ky}\right)\\ M_{y}=\sum_{k=1}^{4}\left(M_{ky}+F_{kx}d_{kz}+F_{kz}d_{kx}\right)\\ M_{z}=\sum_{k=1}^{4}\left(M_{kz}+F_{kx}d_{ky}+F_{ky}d_{kx}\right) \end{array}\right.$$ (24)

where *M*_*x*_, *M*_*y*_, and *M*_*z*_ are three coordinate components of swinging moment. *M*_*kx*_, *M*_*ky*_, and *M*_*kz*_ are three coordinate components of reaction moment at the link pair *k*. *d*_*kx*_, *d*_*ky*_, and *d*_*kz*_ are coordinate components of vector connecting the frame pair k and the motion pair O_1_.

**Fig 10:**
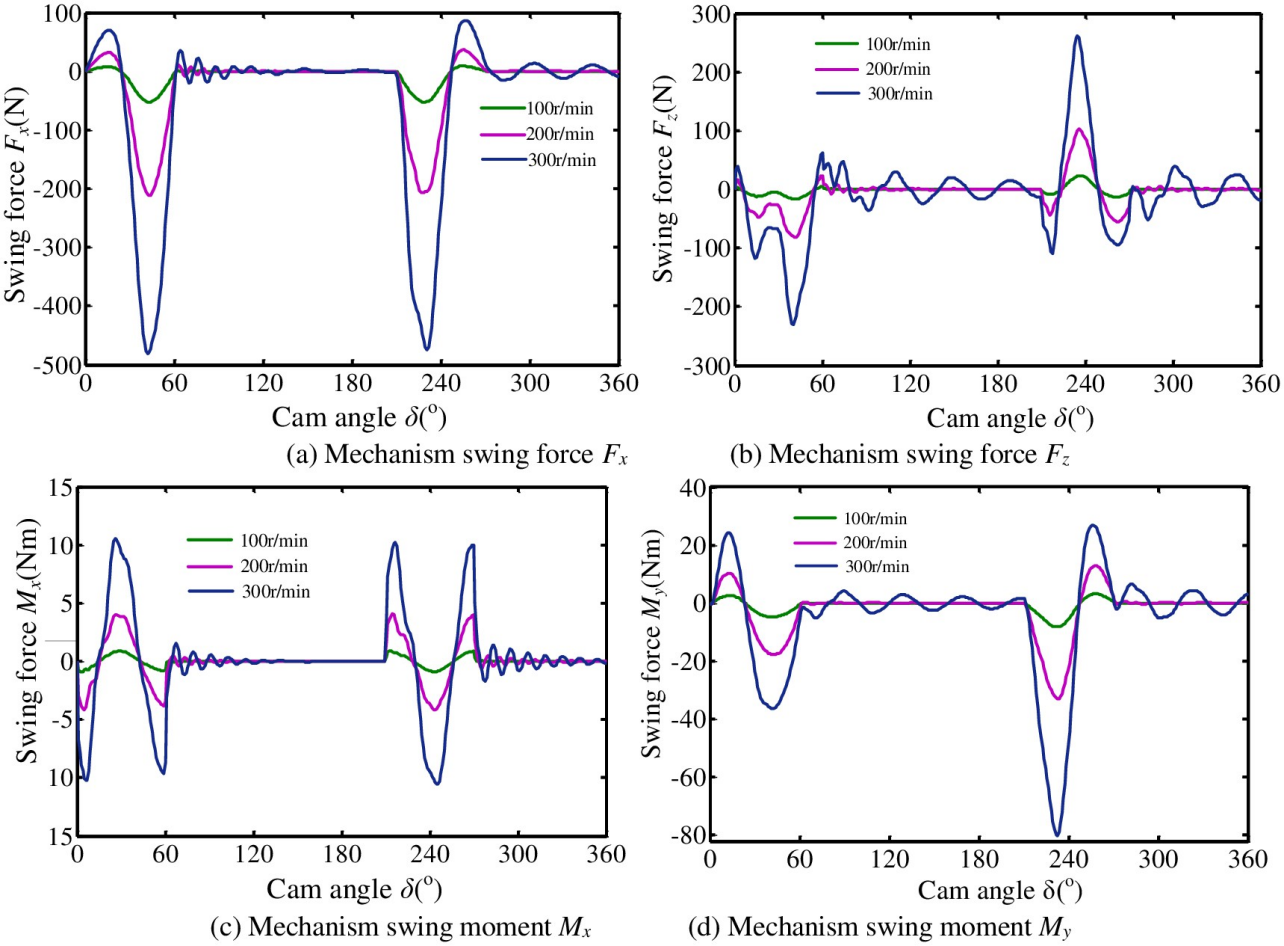
Swing force (swing torque) of the overturning mechanism. (a) Mechanism swing force *F*_*x*_. (b) Mechanism swing force *F*_*z*_. (c) Mechanism swing moment *M*_*x*_. (d) Mechanism swing moment *M*_*y*_.

The movement of pendulum rods O_1_B_1_, O_2_B_2_ and A_1_A_2_ in the machine turning mechanism is mainly the plane movement in *x*O_1_*z*. And the movement range of space links B_1_C_1_ and B_2_C_2_ is also relatively small. Therefore, the swinging force in the *y*-axis direction and the swinging moment in the *z*-axis are relatively small. This article will not analyze it. The swing force and swing moment of flip mechanism at different speeds are shown in Fig 10. The elastic deformation of overturning mechanism components makes the swinging moment of the mechanism produce severe reciprocating oscillations. It is easy to cause severe vibration of the mechanism on the frame and aggravate the impact wear at the kinematic pair and reduce the reliability of the mechanism. It can also be seen from figures that rotational speed and cam profile are important factors affecting the performance of the mechanism.

## 7 Optimal design of flipping mechanism

### 7.1 Optimization of connecting rod section size

Length parameters and section parameters are two important structural parameters of linkage components. The length parameter is determined by rigid body motion of the mechanism. In the case that the motion law of the mechanism has been determined, length parameters of components should not be changed. At this time, changing section parameters of the component becomes an effective way to improve the dynamic performance of the mechanism. In this paper, the influence of cross-section parameters of pendulum rod O_1_B_1_, O_2_B_2_ and connecting rod B_1_C_1_ on the dynamic performance of shear mechanism will be studied respectively. In the research process, the dynamic stress of the mechanism components will be discussed as an example.

#### 7.1.1 Influence of sectional dimension of pendulum rod O_1_B_1_

Pendulum rod O_1_B_1_ is a rectangular cross-sectional member. Its cross-sectional parameters include two aspects: cross-sectional size and cross-sectional shape. First, under the condition that the shape of section remains unchanged (the aspect ratio of section remains unchanged), the effect of section size on the dynamic performance of turning mechanism is studied. Keep the section length-width ratio *λ* = *a*/*b* = 2 of pendulum rod O_1_B_1_ unchanged. Section width *b* varies from 4mm to 18mm. The influence of cross-sectional size of pendulum rod O_1_B_1_ on the performance of the turning mechanism is analyzed.

**Fig 11:**
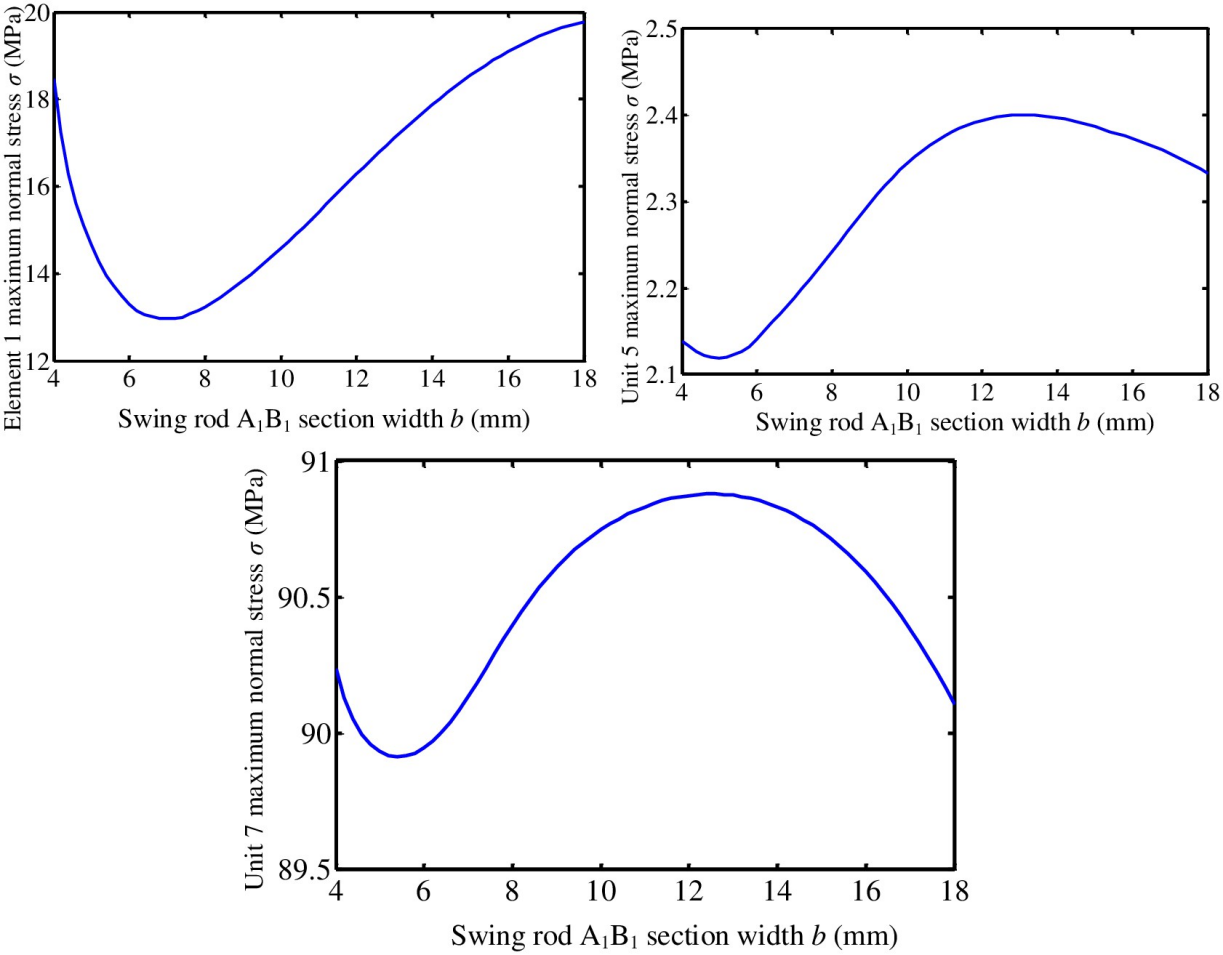
The dynamic stress of the mechanism varies with width b of A_1_B_1_ section.

Fig 11 shows curve of the change of maximum normal stress of turning mechanism unit 1, unit 5, and unit 7 with the section width *b* of pendulum rod O_1_B_1_, respectively. With the continuous increase of section width *b*, the dynamic stress of components fluctuates to a certain extent. The dynamic stress of the unit is the smallest in the range of 6-8mm section width of pendulum rod A_1_B_1_.

**(1) Influence of O**_**2**_**B**_**2**_ **section parameters of the pendulum rod**. Keep length-width ratio *λ* = *a*/*b* = 2 of O_2_B_2_ section of the pendulum rod unchanged. Section width b varies from 4mm to 18mm. The influence of O_2_B_2_ section size of the pendulum rod on the performance of turning mechanism is analyzed.

**Fig 12:**
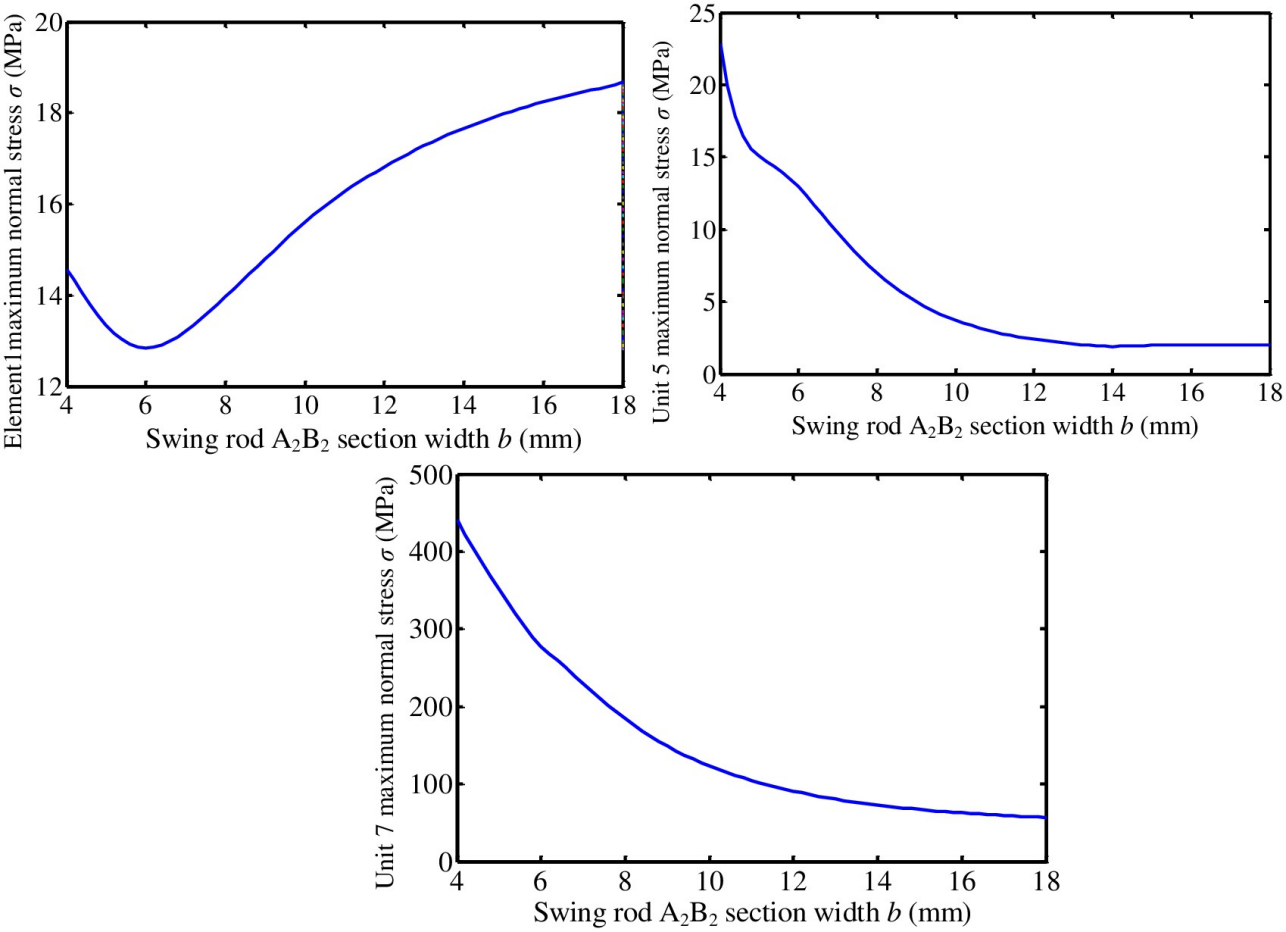
The dynamic stress of the mechanism varies with width b of the A_1_B_1_ section.

Fig 12 shows change the curves of maximum normal stress of turning mechanism unit 1, unit 5, and unit 7 respectively with cross-sectional width *b* of pendulum rod O_2_B_2_. The dynamic stress of the unit is the smallest in the range of 8-10mm in the section width of pendulum rod A_1_B_1_.

**Fig 13:**
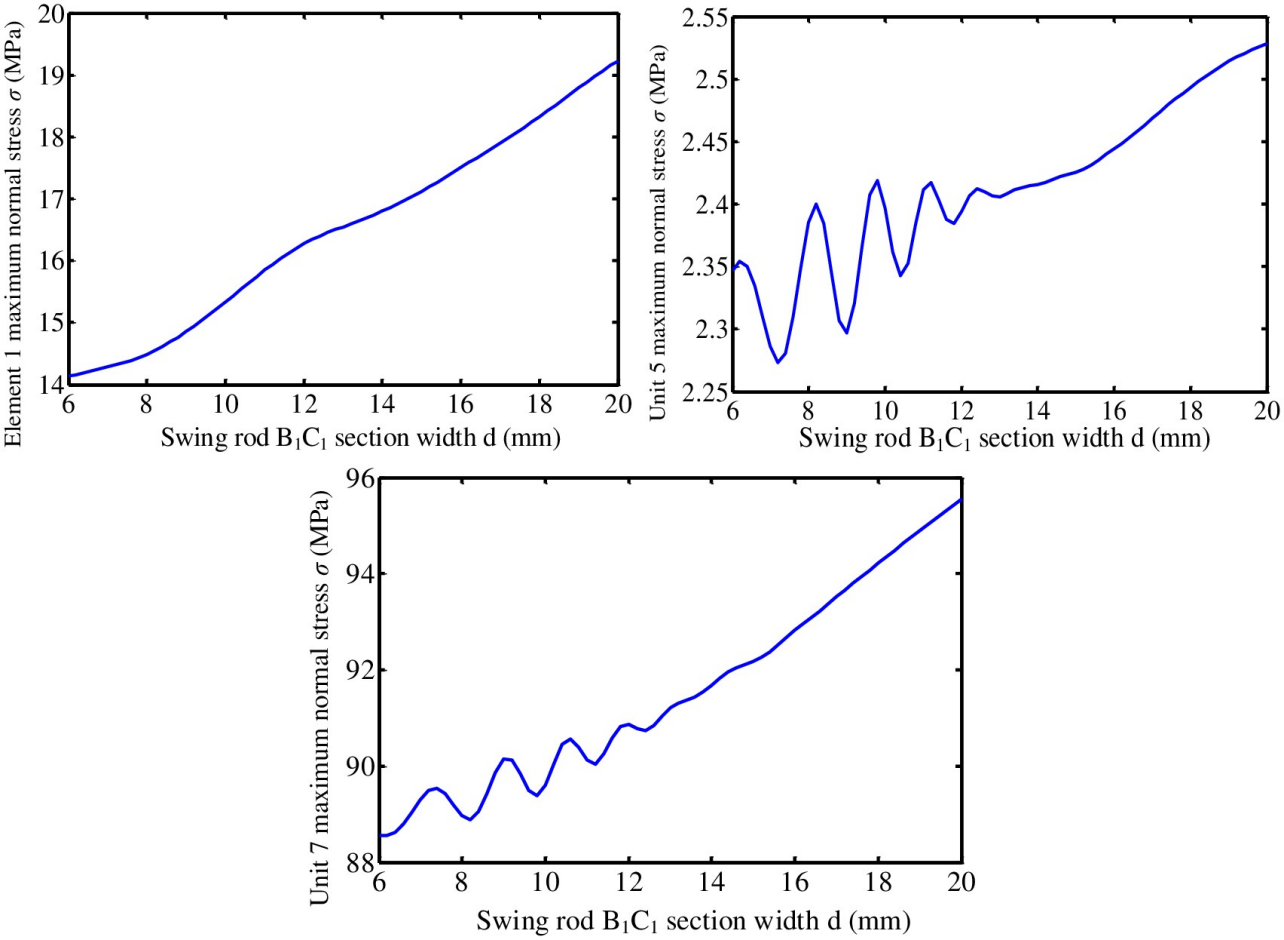
The dynamic stress of the mechanism varies with diameter d of B_1_C_1_.

**(2) Influence of connecting rod B**_**1**_**C**_**1**_ **section parameters**. Connecting rod B_1_C_1_ is a circular section member. Change the diameter of connecting rod B_1_C_1_ section from 6 to 20. The influence of B_1_C_1_ section parameters of connecting rod on the dynamic performance of the mechanism is studied. Fig 13 shows the variation curve of maximum normal stress of turning mechanism unit 1, unit 5, and unit 7 with the diameter *d* of connecting rod B_1_C_1_. The maximum normal stress of three elements increases with the increase of diameter of connecting rod B_1_C_1_. When the diameter of connecting rod changes in the interval [6, 12], maximum stress of element 5 and element 7 fluctuates greatly.

### 7.2 Optimal design of cam structure

The cam mechanism has the characteristics of compact structure, high wear resistance and high reliability. It is widely used in various automatic machinery. The design of motion law of the follower is an important content in the design of cam mechanism. The key to optimal design of cam mechanism is to parameterize the motion law of the follower and select appropriate design variables. There are many design methods for cam profile curves, such as B-spline curves, Bezier curves, classical line curves, Hermitian curves, Schumacher quadratic curves, etc. Classic splines are composed of polylines. It is flexible, adaptable and easy to apply.

#### 7.2.1 Cam profile optimization

In order to ensure the jerk of cam profile is continuous. In this paper, B-spline curve is used as the motion law of a cam follower to study the optimal design of the cam profile.

(1) The law of motion of B-spline curve.

**Fig 14:**
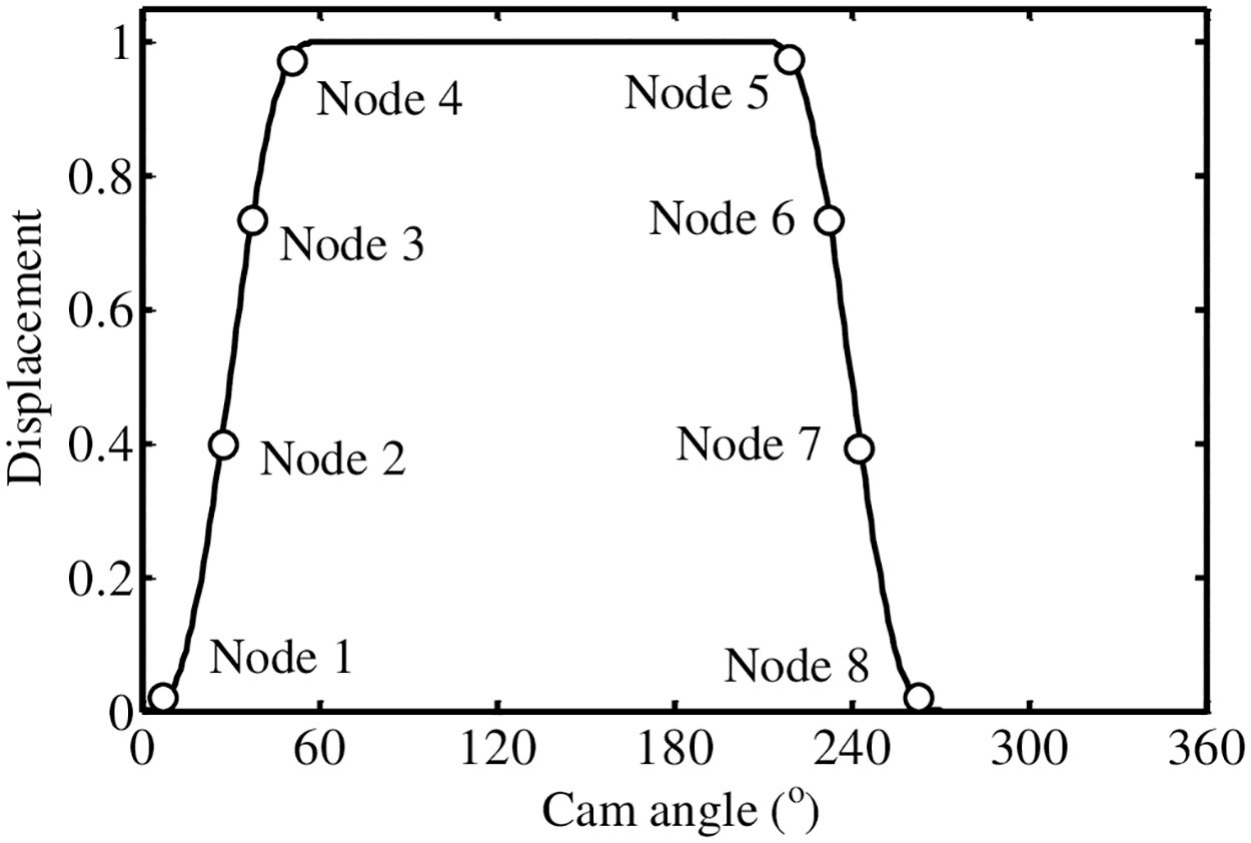
Follower displacement and nodal distribution.

*(1) The law of motion of B-spline curve*. The *k*-order B-spline curve is *C*^*k*-*r*^ continuous at the node with the repetition degree *r*. In order to ensure the continuity of the jerk curve of cam follower’s motion law, fifth-order B-spline curve is used to design follower’s motion law. The motion law and node position of a cam follower of flip mechanism are shown in Fig 14. The node sequence is symmetrically distributed and the repetition degree of node 1, node 4 and node 5, node 8 is set to 6. The node sequence of a cam follower displacement curve is as follows:

Follower lift motion phase: *t*_1_ = … = *t*_6_ = 0, *t*_7_ = *θ*_*t*1_, *t*_8_ = *θ*_*t*2_, *t*_9_ = … = *t*_14_ = *π*/3,
Follower return movement phase: *t*_15_ = … = *t*_20_ = 7*π*/6, *t*_21_ = 3*π*/2-*θ*_*t*2_, *t*_22_ = 3*π*/2-*θ*_*t*1_, *t*_23_ = … = *t*_28_ = 3*π*/2

According to the node distribution of a follower displacement curve, the displacement expressions of cams lift and return phases can be obtained:

$$\left\{\begin{array}{l} s_{升}\left(\theta \right)=\sum_{i=1}^{8}a_{i}N_{i,5}\left(\theta \right)\\ s_{回}\left(\theta \right)=\sum_{j=1}^{8}a_{j}N_{j,5}\left(\theta \right) \end{array}\right.$$ (25)

In the formula, *a*_*i*_, *a*_*j*_ (*i*, *j* = 1, 2,…,8) are control parameters, which are determined by the motion constraints (4) of the follower motion law:

$$\left\{\begin{array}{l} s\left(0\right)=0,s^{\prime }\left(0\right)=0,s^{\prime \prime }\left(0\right)=0,s\left(\theta_{t1}\right)=s\left(\frac{5\pi }{9}-\theta_{t1}\right)=s_{t1},s\left(\frac{5\pi }{18}\right)=s_{m}\\ s\left(\theta_{t2}\right)=s\left(\frac{5\pi }{9}-\theta_{t2}\right)=s_{t2},s\left(\frac{5\pi }{9}\right)=0,s^{\prime }\left(\frac{5\pi }{9}\right)=0,s^{\prime \prime }\left(\frac{5\pi }{9}\right)=0 \end{array}\right.$$ (26)

#### 7.2.2 Establishment of variable constraints for optimal design of flip mechanisms

*(1) Objective function and design variables*. The swing force (swing moment) is main cause of the vibration of a mechanism on the frame. The swing force and swing moment of the mechanism are selected as objective functions of the optimization model, Expression is shown in (27):

$$V-\text{min}\;f=\text{min}{\left[\left|F\right|\;\left|M\right|\right]}^{T}$$ (27)

Eq (27) is a multi-objective optimization design problem in which maximum value of swing force and swing moment in one motion cycle of flipping mechanism is the minimum. *F* is the total swinging force of shearing mechanism. *M* is the total swinging moment of the mechanism. And expressions of *F* and *M* are shown in formula (28):

$$\left\{\begin{array}{l} F=\sqrt{F_{x}^{2}+F_{z}^{2}}\\ M=\sqrt{M_{x}^{2}+M_{y}^{2}} \end{array}\right.$$ (28)

In formula (28), *F*_*x*_ and *F*_*z*_ are the coordinate components of the swing force of the mechanism; *M*_*x*_ and *M*_*y*_ are the coordinate components of the swing moment. The components of the overturning mechanism are mainly based on the movement in the *xoz* plane. The values of coordinate components *F*_*y*_ and *M*_*z*_ of swing force and swing moment are relatively small, so they are no longer considered in the mechanism optimization model.

In order to optimize each component of objective function. When solving the multi-objective optimization problem, another evaluation function is constructed according to the specific problem of multi-objective optimization. A multi-objective optimization problem is transformed into a single-objective optimization problem that solves evaluation function. In the transformation, linear weighted sum method can be used, that is, the evaluation function is constructed by assigning appropriate weight coefficients to each sub-objective function. Using a linear weighted sum method, the objective function of flip mechanism optimization model can be expressed as follows:

$$f=w_{1}f_{1}+w_{2}f_{2}$$ (29)

The core of the linear weighted sum method is to reasonably determine the weight coefficient *w*_*i*_ of sub-objective function. Two sub-objective functions in the optimization model of flip mechanism can determine the weight coefficient of the sub-objective function according to the formula (30):

$$w_{1}=1/f_{1}^{*},w_{2}=1/f_{2}^{*}$$ (30)

In the formula, $f_{1}^{*}=\text{min}\;f_{1}\left(x\right)$ is the optimal value of swing force of a mechanism, $f_{2}^{*}=\text{min}\;f_{2}\left(x\right)$ is the optimal value of the swinging moment of shearing mechanism. Objective functions determined by Eqs (29) and (30) reflect degrees to which each single objective function deviates from its respective optimal value.

The length of a connecting rod in the flipping mechanism is determined by expected motion law of the end effector. Therefore, cross-section parameters of a connecting rod are selected as design variables in terms of structural parameters of connecting rods. For the optimization of the cam profile of flip mechanism, node parameters of the motion law of B-spline curve are selected as design variables. Design variables of mechanism optimization models are written in vector form as shown in formula (31):

$$x=\left[\begin{array}{ccccccc} \theta_{t1} & s_{t1} & \theta_{t2} & s_{t2} & b & \lambda & d \end{array}\right]$$ (31)

In the formula, *θ*_*t*1_, *s*_*t*1_ and *θ*_*t*2_, *s*_*t*2_ are node parameters of the motion law of B-spline curve. *b* is the cross-sectional width of pendulum rod O_1_B_1_ and pendulum rod O_2_B_2_. *λ* is the length-width ratio of the cross-section of pendulum rods O_1_B_1_ and O_2_B_2_. *d* represents the diameter of connecting rods B_1_C_1_ and B_2_C_2_.

The dynamic stress of the mechanism should be limited during the optimization process of flip mechanism. Cam profile of the mechanism should also meet some constraints such as processing technology and strength requirements. The constraints of the mechanism optimization model are shown in formula (32):

$$\left\{\begin{array}{ll} \sigma_{AB}\leq \left[\sigma \right],\sigma_{BC}\leq \left[\sigma \right],\sigma_{DE}\leq \left[\sigma \right] & \left(a\right)\\ \left|\rho \right|\leq r_{r},\alpha_{\text{max}}\leq \left[\alpha \right] & \left(b\right)\\ \sigma_{H\text{max}}\leq \left[\sigma_{H}\right],F_{\text{min}}\geq 0 & \left(c\right) \end{array}\right.$$ (32)

In formula (32), formula (*a)* is the dynamic stress constraint condition of the mechanism, which is used to limit the dynamic stress of mechanism; (b) is the geometric constraint of cam profile, which is used to limit the minimum radius of curvature of the cam profile to prevent overcut during the cam manufacturing process; (c) Strength constraint mainly limits the contact stress between cam rollers.

#### 7.2.3 Comparative analysis of simulation results

**Fig 15:**
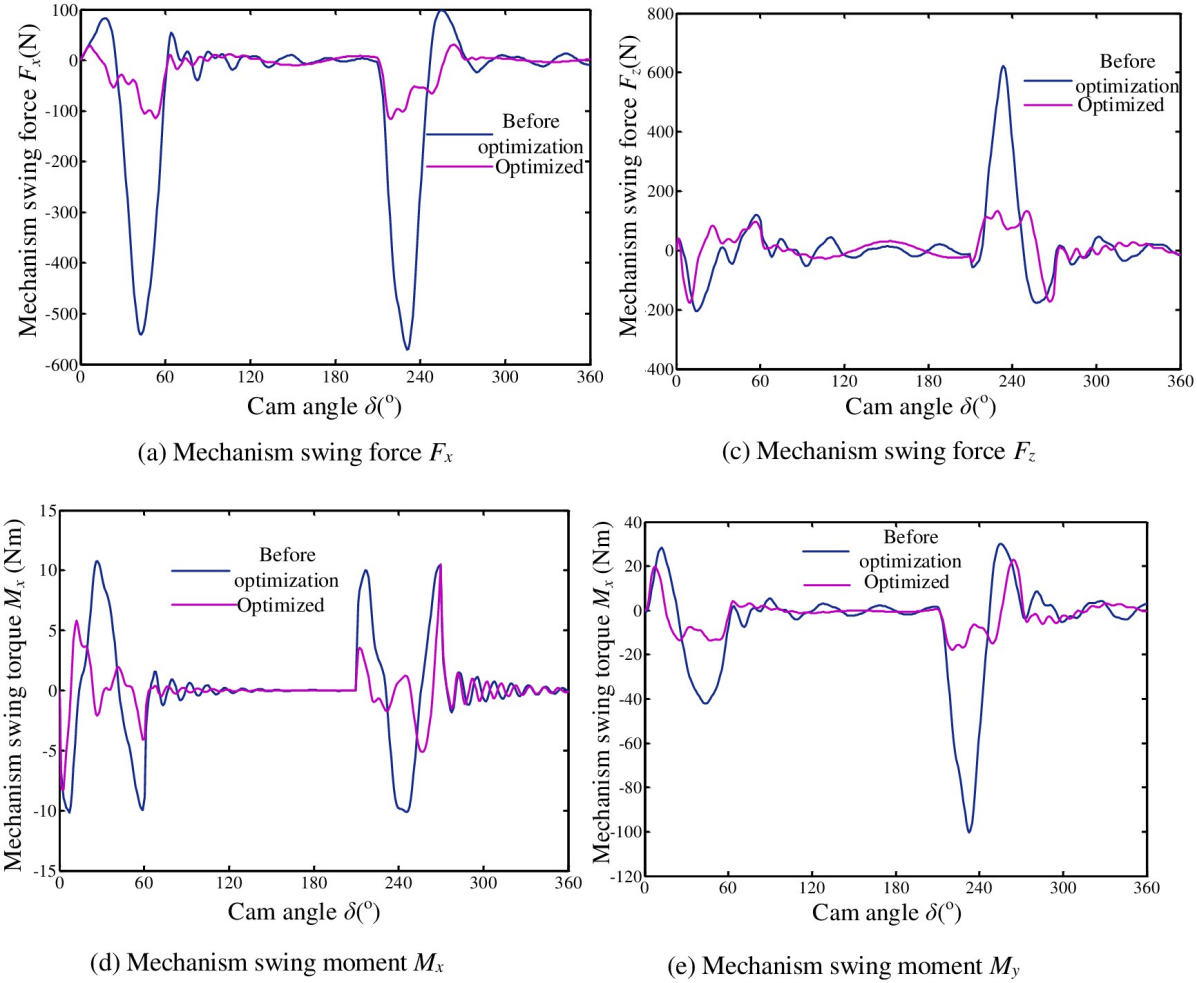
The change curve of the swing force (swing moment) of the mechanism. (a) Mechanism swing force *F*_*x*_. (b) Mechanism swing force *F*_*z*_. (c) Mechanism swing moment *M*_*x*_.

In order to ensure that the desired result is the global optimum, this paper selects genetic algorithm as solution methods for an optimization model of flipping mechanism [29]. It is necessary to transform the practical problem to be solved from modeling space to the programming space when the genetic algorithm is applied. According to the theoretical model in Section 3 and the optimization model in this chapter. Compile objective function m-file and combine with MATLAB genetic algorithm toolbox. Solve the optimization model and get the optimal solution. Optimization results are shown in Fig 15. It can be seen from the figure that after optimization, two main components of the mechanism swing force and swing torque have a relatively large decrease. The residual vibration amplitude of mechanisms at rest stage is much lower than that before optimization. A curve becomes smoother. In order to intuitively compare the changes of various dynamic indicators of a mechanism before and after optimization. The maximum value and variation range of the swing force, swing moment and link dynamic stress of mechanisms before and after optimization are listed in Table 1.

**Table 1:** Optimization results of dynamic parameters of flipping mechanism.

content	*F*_*x*_ (N)	*F*_*y*_ (N)	*M*_*x*_ (Nm)	*M*_*y*_ (Nm)	*σ*_*O*1*B*1_ (MPa)	*σ*_*O*2*B*2_ (MPa)	*σ*_*B*1*C*1_ (MPa)	*σ*_*B*2*C*2_ (MPa)
Before optimization	571.5	621.2	10.8	100.4	16.3	10.8	32.7	90.9
Optimized	115.9	176	10.5	23	6.2	4.1	37.9	100.2
Amplitude/%	79.7	71.7	2.8	77.1	62	62	-15.9	-10.2

It can be seen from Table 1 that for different kinetic indicators, the optimization effect is different. Among coordinate components of the swing force and swing moment, the component with the larger maximum value also has the largest decrease after optimization. After the mechanism is optimized, not only the swing force and swing moment are improved, but also the dynamic stress of some connecting rods is greatly reduced. This shows that it is reasonable and effective to choose the swing force and swing moment of the mechanism as objective functions of the mechanism optimization. The data in Table 1 show that the maximum dynamic stress of connecting rods B_1_C_1_ and B_2_C_2_ not only did not improve but also increased in amplitude. This is mainly because the dynamic stress of the component is closely related to the force of the mechanism and the cross-sectional parameters of the component. Due to the mechanism optimization model, connecting rods section parameters are also selected as design variables. The design variables after mechanism optimization are: ***x*** = [0.34 0.32 0.55 0.63 9.8 2.1 6.4]^*T*^. The structural parameters of connecting rods before and after optimization do not change much, which shows that the optimization of cam profiles has played a great role in improving the dynamic characteristics of the mechanism.

## Conclusions

First, this paper establishes the kinematic equations of the flipping mechanism. The relationship between connecting rods, slider displacement and cam rotation angles of flipping mechanism is analyzed. It can be seen that space link mechanism not only plays the function of motion transmission, but also amplifies the input motion of cam pendulum mechanism. Therefore, the rational design of a cam profile curve is the key to solving this problem.

Second, we use the finite element method to establish the metadynamic equation of the spatial linkage subsystem. The dynamic equation of the cam roller system is constructed by the lumped parameter method and solved by the Newmark method. The variation law of elastic displacement with cam rotation angle in different ranges of cam speed from 100r/min to 300r/min is analyzed. The research shows that the rotational speed of camshaft is a factor that has the greatest influence on the elastic dynamic characteristics of the mechanism. It is necessary to further analyze optimal parameters of connecting rods and cam profile curves of the mechanism with the help of dynamic equations.

Third, we found that the actuator of the rotating mechanism was changed from a connecting rod mechanism to a rack and pinion, which would make the operation more stable and reliable. The swing force (moment) of the mechanism has been improved to a certain extent. But the degree of improvement of different components is not the same. And a cam profile curve and transmission part have a great influence on the dynamic characteristics of the mechanism. The comparison of two improves the reliability of a flipping mechanism system and reduces the adverse effects of the elastic movement of the link on the flipping mechanism system at high speed. The optimized design model of the flipped plastic surgery mechanism was solved and the optimization results were obtained. Completed the optimal design of the flipped shaping mechanism. Therefore, the adoption of this method will optimize the mechanical mechanism of the equipment and reduce vibration. At the same time, it improves the stability of the equipment under high-speed operation.

## Supporting information

S1 Appendix (DOCX)
