# Supplementary material for: Design and optimization of complex mechanism flip shaping subsystem based on genetic algorithm and rigid-flexible coupled dynamic model
Source: PLoS One. 2023 Feb 2;18(2):e0280983. doi: 10.1371/journal.pone.0280983 (PMC9894479; doi:10.1371/journal.pone.0280983)
Supplement: S1 Appendix — (DOCX) [file pone.0280983.s001.docx]

**Appendix**

**Notation**

| $a_{\begin{matrix} i \\ \end{matrix}}$ | weighting parameters of B-spline basis function |
| --- | --- |
| $a_{i}(i=1,2,...,11)$ | control parameter determined by motion constraint |
| $B_{i}^{T}$ | multiplied by the right side of the equation |
| $c$ | contact damping coefficient |
| $c{}_{cy}$,$c_{cx}$ | projections of contact damping coefficient between cam and roller |
| $c_{f}$ | friction damping coefficient of camshaft support bearing |
| $c{}_{x}$,$c_{y}$ | corresponding damping coefficients |
| $d{}_{kx}$,$d_{ky}$,$d_{kz}$ | coordinate components of connection vector between connecting rack pair *k* |
| $E_{1}$，$E_{2}$ | material elastic modulus of cam and roller |
| $F$ | positive pressure |
| $F$ | generalized external force array |
| $F_{ei}$ | generalized external force |
| $F_{kx}$，$F_{ky}$，$F_{kz}$ | three coordinate components of the reaction of motion pairs at connecting rack pair *k* |
| $F_{x}$，$F_{y}$ | interaction effects between roller and swing lever OA |
| $h_{1}$,$h_{2}$ | depth of contact deformations on roller and cam |
| $J_{e}$ | equivalent moment of inertia of planar four-bar mechanism |
| $k$ | contact stiffness |
| $k_{c}$ | contact stiffness between cam and roller |
| $k_{cx}$,$k_{cy}$ | projections of contact stiffness |
| $k_{x}$,$k_{y}$ | bending stiffness of camshaft |
| $\bar{K}$ | effective rigid matrix of the system |
| $m_{1}$,$m_{2}$ | related mass parameters |
| $m_{1}$,$m_{2}$ | lumped masses of cam and roller |
| $M_{C}$ | moment of linking rod BC on swing lever CD |
| $M_{kx}$,$M_{ky}$,$M_{kz}$ | three coordinate components of counter moment at connecting rack pair *k* |
| $M_{L}$ | mass matrix |
| $M_{x}$,$M_{y}$,$M_{z}$ | three coordinate components of shaking moment |
| $P_{ei}$ | inertia force the element suffered |
| $Q$ | internal force of linkage subsystem |
| $Q_{ei}$ | reaction of the kinematic pair |
| $R_{1}$,$R_{2}$ | radius of curvature of the contact area |
| $U_{i}$ | generalized coordinate array of element *i* |
| $U^{*}$ | coordinate matrix $B_{i}$ |
| $U_{60}$ | elastic rotation angle of DE in Node D |
| $U_{62}$ | elastic rotation angle of linking rod DE in Node E |
| $\beta_{1}$,$\beta_{2}$,$\beta_{4}$ | swing angles of swing rod $O_{3}B_{3}$,$C_{3}D_{3}$ |
| $\Delta t$ | time step |
| $\lambda$ | ratio of length and width of section |
| $\mu_{1}$,$\mu_{2}$ | material Poisson ratios of cam and roller |
| $\mu_{1}$,$\mu_{2}$ | solving parameters |
| $\xi$ | damping ratio |
| $B_{i}$ | coordination matrix of 8 × *Nu* and generated $I_{u}$ according to the model composition matrix |
| $E$ | elastic modulus of the beam element |
| $F_{i}^{e}$ | includes the external force and the unit interaction force |
| $h$ | height distance between the neutral layer of the beam element section and its outer surface |
| $J_{1}$ | rotation inertia of the rod AB around point A. |
| $K$ | mass matrix |
| $M$ | a linear combination of the mass matrix |
| $P$ | refers to design variable array |
| $U$ | system of independent generalized coordinate |
| $U^{e}$ | unit in the generalized coordinates of the number |
|  | array dimension |
| $Ui$ | direction of elastic displacement for a certain *x*-axis or *y*-axis |
|  | corresponding rigid body acceleration for x-axis or *y*-axis |
|  | rigid body acceleration at the node i in the *x*-axis |
| $\gamma_{1}$，$\gamma_{2}$ | weight coefficient |
| $\xi_{i}$，$\xi_{j}$ | damping ratio |
| $\eta$，$\lambda$ | damping parameters |
|  | rigid corner angular velocity of the component *i* |
| $\theta_{1}$，$\theta_{2}$，$\theta_{3}$ | angle between the rod AB, rod BC, rod CD and *x*-axis |
| $\omega_{i}$ | natural frequency of any *i* order of the system |
| $\omega_{j}$ | natural frequency of any *j* order of the system |
